# LMO7-mediated POLR2A degradation promotes cellular senescence through the MDM4/p53/p21 axis

**DOI:** 10.1038/s41419-026-08679-0

**Published:** 2026-03-28

**Authors:** Chutong Lai, Wen Fu, Jiaxin Liu, Shuai Hou, Jinsong Yan, Haixin Lei

**Affiliations:** 1 Institute of Cancer Stem Cell, Dalian Medical University, Dalian, PR China; 2 Liaoning Provincial Key Laboratory of Nucleic Acids Biology, Dalian Medical University, Dalian, PR China; 3 Department of Hematology, Liaoning Medical Center for Hematopoietic Stem Cell Trans-plantation, The Second Hospital of Dalian Medical University, Dalian, PR China; 4 College of Basic Medical Sciences, Hainan Medical University, Haikou, PR China

**Keywords:** Senescence, Ubiquitylation, Transcription

## Abstract

As the largest subunit of RNA polymerase II, POLR2A plays an irreplaceable role in gene expression, with the regulation of its own expression and physiological function having attracted widespread attention. Here we report POLR2A as a critical guardian against cellular senescence. A significant decline in POLR2A expression was observed in senescent cells and certain tissues of aging mice. Whereas its depletion dramatically induced cellular senescence, conversely, activating endogenous POLR2A expression in senescent cells using CRISPRa technology alleviated the senescent phenotype. We further demonstrated that POLR2A-induced senescence is p53-dependent, as evidenced by the activation of the p53/p21 pathway upon POLR2A knockdown and the rescue of the senescence phenotype following co-depletion of POLR2A and p53. Bioinformatic analysis on RNA-seq data from POLR2A depletion and replicative senescent cells led to the identification of MDM4 as the key mediator of p53 upregulation following POLR2A knockdown. Most intriguingly, immunoprecipitation assay further revealed that the E3 ligase LMO7 was recruited to POLR2A to promote the ubiquitination and proteasomal degradation of POLR2A under cellular senescence. Depletion of LMO7 abolished the ubiquitination and reduction of POLR2A in H₂O₂-induced senescent cells. Taken together, we concluded that the LMO7-induced POLR2A degradation drived cellular senescence through the MDM4/p53/p21 axis.

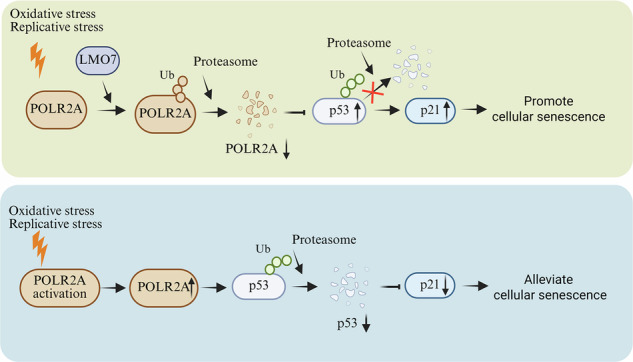

## Introduction

RNA polymerase II (Pol II) is responsible for transcribing all protein-coding genes as well as numerous non-coding RNA genes in eukaryotes. Dysregulated Pol II-driven transcription has been implicated in cancer and aging process [1–3]. As the largest and key catalytic subunit of Pol II, POLR2A plays an irreplaceable role in gene expression. However, relatively little attention has been given to the regulation of its own expression, with existing studies largely centred on ubiquitin-mediated degradation mechanisms of the POLR2A protein under conditions such as DNA damage or Pol II stalling [4–6]. POLR2A ubiquitination is a complex and multistep process mediated by a variety of distinct E3 ubiquitin ligases, such as Nedd4, WWP2, CRL4^CSA^ [7–9]. Recently, it has been reported that ARMC5, CUL3, and RBX1 form an activated E3 ligase complex, which is mainly responsible for POLR2A ubiquitination in normal cells and tissues [10]. However, the mechanism of POLR2A protein degradation under other conditions is not yet known.

Recent studies have demonstrated that POLR2A is closely associated with many diseases including cancer [11, 12]. Moreover, the inability of cells to degrade aberrantly stalled Pol II constitutes a major pathogenic mechanism underlying Cockayne syndrome, a premature aging disease [13]. In addition, inhibition of DNA damage-induced POLR2A ubiquitination has been shown to promote premature aging in mice [9]. A comprehensive proteomic and histological analysis recently revealed the significant downregulation of POLR2A in aged human tissues, including aorta, spleen, and adrenal gland [14]. Cellular senescence, a state of irreversible growth arrest triggered by diverse stresses, represents a hallmark of aging [15]. It has been shown that POLR2A expression is significantly downregulated in both Werner syndrome patients and normal old donor cells [16]. Furthermore, a previous report from our laboratory demonstrated that knockdown of POLR2A induced dramatic cellular senescence in human skin fibroblast HFF1 cells, indicating a potential role of POLR2A in the regulation of cellular senescence [17]. However, the precise role and mechanism in regulating cellular senescence have not been elucidated.

The most classically studied pathways involved in the regulation of cellular senescence are p53/p21^CIP1^ and p16^INK4a^/RB pathways [18]. The p21^CIP1^ activation occurs primarily in the initial phase of senescence, while the p16^INK4a^ is induced primarily at later phase and contributes to the maintenance of cellular senescence [18]. Under senescence stressors, p53 is activated through either DNA damage response (DDR)-dependent or DDR-independent pathways, leading to the transcriptional upregulation of its downstream target p21^CIP1^ [18]. The activation of p53 is tightly regulated by different factors at different levels [18]. In terms of post-translational modifications, ubiquitination plays an important role in regulating p53 level and activity. The E3 ubiquitin ligase MDM2 and its family member MDM4 are the most well-known negative regulators of p53 [19, 20]. A group of other E3 ligases, including UBE4B, TRIM24, TRIM28, TRIM69, TRIM39, FBXW7, and Pirh2, have also been reported to ubiquitinate p53 and promote its degradation [21–27]. Another group, such as WWP1, E4F1, Ubc13, and MSL2, catalyze p53 ubiquitination without triggering proteasomal degradation, thereby regulating its function through alternative mechanisms[28]. Preliminary studies in our lab have found that downregulation of POLR2A in HFF1 cells led to p53/p21 pathway activation, but the exact mechanism remains to be elucidated [17].

In this study, we demonstrated that POLR2A protein was significantly downregulated in senescent cells using both replicative and hydrogen peroxide-induced cellular senescence models as well as aged mouse model in vivo. Furthermore, we identified that POLR2A depletion induced cellular senescence through activation of p53/p21 pathway mediated by MDM4. In particular, we discovered that the downregulation of POLR2A protein in senescent cells resulted from LMO7-mediated ubiquitination and proteasomal degradation of POLR2A protein. Finally, activation of endogenous POLR2A expression in senescent cells by CRISPRa technology alleviated cellular senescence. Taken together, this study indicated a novel LMO7/POLR2A/MDM4/p53/p21 axis as a critical regulator of cellular senescence.

## Material and methods

### Cell culture

HFF1, MRC5 and 293 T cells were purchased from the American Type Culture Collection. HFF1 cells were cultured in DMEM medium with 15% FBS (VivaCell, China) and 1% P/S, MRC5 was cultured in MEM medium supplemented with 10% FBS and 1% P/S, and 293 T was cultured in DMEM medium supplemented with 10% FBS and 1% P/S. All cells were cultured at 37 °C in a humidified incubator with 5% CO_2_.

### siRNA knockdown

For siRNA-mediated knockdown, HFF1 or MRC5 cells were inoculated in six-well plates, and 2 μL siRNAs (50 μM) were transfected with Lipofectamine 3000 when the cell density reached 30%-40%. Cells were harvested at 48, 72, or 96 h, depending on the experimental requirements. All siRNAs were synthesized by RiboBio (China), and the target sequences were shown in Supplementary Table S1.

### Inhibitor and H_2_O_2_ treatment

To induce cellular senescence, HFF1 or MRC5 cells were cultured in 6-well plates. When the cells reached ~30% confluency, they were treated with 200 μM or 400 μM H_2_O_2_ for 4 h, respectively. Then the medium was replaced with fresh complete medium and the incubation was continued for 96 h.

To investigate the effect of POLR2A knockdown on p53 protein stability, 60 h after knockdown of POLR2A in HFF1 cells, the cells were treated with 50 µg/mL cycloheximide (CHX) for 0, 1, 1.5, and 2 h, respectively, and subsequently collected.

To test whether the reduction of POLR2A protein in H_2_O_2_-induced senescent cells is dependent on the proteasomal pathway, HFF1 cells were treated with 200 μM H_2_O_2_ for 4 h, then the medium was replaced with fresh complete medium, and incubation was continued for 72 h. Then, the cells were added with 20 µg/mL MG132, and harvested at 0, 12 and 24 h, respectively.

To inhibit the ubiquitination and degradation of POLR2A protein during cellular senescence, HFF1 cells were treated with H_2_O_2_ for 4 h, replaced with fresh complete medium, and continued to be cultured for 48 h. Then, 0.5 μM MLN4924 was added, and the cells were harvested at 96 h.

### Antibodies

All antibodies are listed in Supplementary Table S2.

### Plasmid construction

CRISPR activation (CRISPRa)-dCas9-VP64 expression vectors (pMSCV-LTR-dCas9-VP64-BFP, #46912) and single guide RNA (sgRNA) expression vector (pSLQ1651-sgTelomere (F + E), #51024) were kindly provided by Dr Juan Shi at Institute of Basic Medical Sciences of Chinese Academy of Medical Sciences. The primers targeting POLR2A were designed according to the Cold Spring Harbor protocol on the CRISPR-ERA website, the fragment targeting POLR2A was amplified by PCR according to the protocol, and the PCR product was inserted into the sgRNA expression vector at the BsXI and XhoI sites. Sequencing was performed after successful plasmid construction. Primers used for construction are listed in Supplemental Table S3.

### CRISPR/dCas9-mediated gene activation

CRISPRa of POLR2A was performed as described before [29]. To endogenously activate POLR2A expression, sgPOLR2A or CRISPRa plasmids were transfected with Rev, Gag and VSV-G packaging vectors in a 2:2:2:1 ratio into 293 T cells. Viruses were harvested 48 h after transfection.

For the H_2_O_2_-induced cellular senescence model, cells were first infected with CRISPRa viruses and then puromycin-screened. Cells that survived the screening were then infected with the sgPOLR2A viruses and puromycin-treated for 5 days to confirm POLR2A activation before H_2_O_2_ induction. For the replicative senescence model, the same procedure was used to activate POLR2A expression, confirm activation, and subsequently passaging.

### Co-immunoprecipitation (co-IP)

For the H_2_O_2_-induced cellular senescence model, HFF1 cells were treated with 200 μM H_2_O_2_ for 4 h and then replaced with fresh complete medium. Whole cell lysates were harvested at 48 h and 96 h, respectively. For the replicative senescence cell model, HFF1 cells were passaged at 1:3 from generation 22 to 37, and both HFF1 generation 22 cells and generation 37 cells were harvested for whole cell lysates. Cells were lysised on ice for 2 h with IP lysis buffer (20 mM Tris-HCl, pH 7.5, 100 mM NaCl, 50 mM NaF, 0.5% NP-40, and a full protease inhibitor cocktail (MCE)), then centrifuged for 30 minutes at 4 °C at 12,000 × *g*. For endogenous co-IP, lysates (2 mg protein) were precleared with 30 μL of Protein G- PLUS Agarose Beads (Thermo Fisher) for 4 h, and then the supernatant was collected by centrifugation at 2000 rpm for 5 minutes at 4 °C. The supernatant was then incubated with the indicated antibodies for 4 h, followed by an overnight incubation with beads at 4 °C. The immunoprecipitated complexes were washed three times with IP Washing buffer (PBS+ proteinase inhibitor cocktail) and then eluted by boiling in 2× SDS loading buffer for 10 min.

### Mass spectrometry (MS) analysis

Protein eluates from IP were separated by 10% SDS-PAGE gel. The gel was stained with the sliver staining kit (Beyotime, China). Subsequently, the gels were cut and sent to the MS platform of Tsinghua University for detection.

### RNA-seq

POLR2A was knocked down in HFF1 cells, and the cells were harvested after 72 h. After SA-β-gal staining to confirm cellular senescence, total RNA from siPOLR2A and siNC cells was extracted and sent to NOVOGENE (China) for detection. First, ribosomal RNA is removed from total RNA. Subsequently, the RNA is fragmented into short fragments of 250–300 bp. Fragmented RNA serves as a template for cDNA synthesis. cDNA fragments around 200 bp are selected. Finally, PCR amplification is performed to obtain the library. For the replicative senescent cell model, HFF1 cells were passaged to 42 generations and assayed in the same manner after confirming cell senescence.

### Chromatin-associated nascent RNA isolation

Chromatin-associated nascent RNA isolation was performed as previously described [17].

### SA-β-gal and EdU staining

SA-β-gal and EdU staining was performed using SA-β-gal staining kit (Beyotime, China) and EdU staining kit (MeilunBio, China) following the manufacturer’s protocols. Cells were then photographed using Ti-S microscope (Nikon) for SA-β-gal staining and DMI8 microscope (Leica) for EdU staining.

### Mice

The animal experimental protocol was approved by the Institutional Animal Care and Use Committee of Dalian Medical University (Approval No. AEE22115). Male C57BL/6 J mice at 1, 9, and 19 months of age were obtained from the SPF Animal Experiment Center of Dalian Medical University. For protein extraction from the heart, brain, lung, and liver tissues, mice were randomly allocated into three groups (*n* = 3 per group) and housed until reaching the target ages. Tissues were harvested, processed into frozen sections, minced, and lysed in RIPA buffer (Solarbio) for 1 h. Following centrifugation, the supernatant proteins were collected for western blot analysis. Blinding was not implemented in this study.

### RT-PCR

Following extraction with TRIzol (Sigma), 1 µg of total RNA was treated with DNase I (Promega) and then reverse transcribed using M-MLV reverse transcriptase (Promega). The resulting cDNA was amplified by PCR, and the products were resolved on an agarose gel. Band intensities were quantified using ImageJ software and normalized against the loading control. The primer sequences were listed in Supplementary Table 3.

### ELISA

After 96-h knockdown of POLR2A in HFF1 cells, the cell culture medium was collected. The concentration of human interleukin 6 (IL-6) and interleukin 8 (IL-8) was measured using an ELISA kit according to the manufacturer’s instructions (Elabscience).

### Statistics

Data from three independent experiments were presented as mean ± SEM (Standard Error of Mean). Statistical analyses between two groups were performed using Student’s t-test with statistical significance defined as: *p < 0.05, **p < 0.01 and ***p < 0.001.

## Results

### POLR2A is downregulated in cellular senescence

**Fig. 1:**
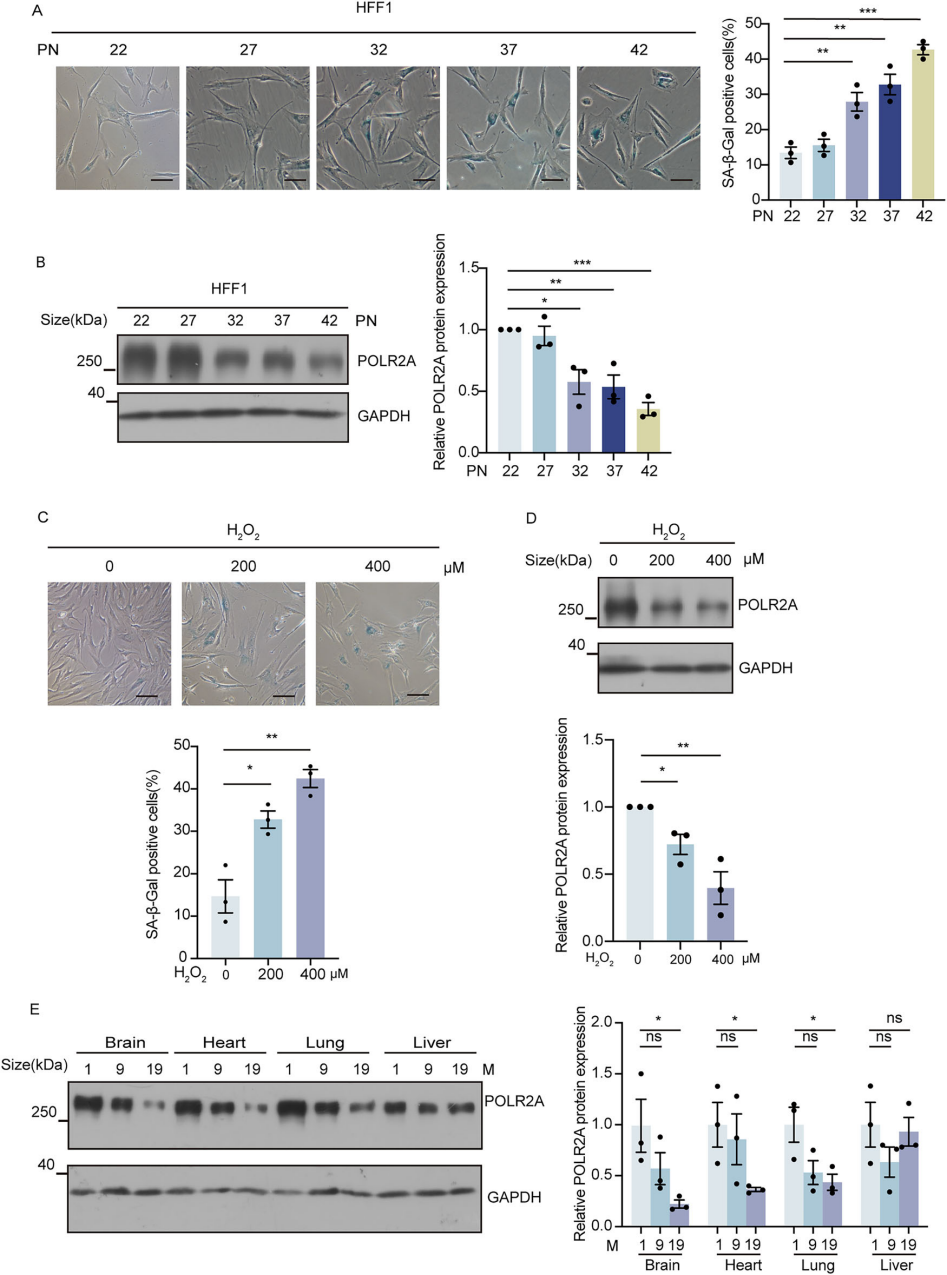
POLR2A expression is reduced in cellular senescence. **A** Representative images of SA-β-gal staining at different passage number (PN) of 22, 27, 32, 37, and 42 in HFF1 cells (the scale bar represents 100 μm). The percentage of SA-β-gal positive (blue) cells had significant increase in late passage (PN 32, 37, 42). *n* = 3. ** *p* < 0.01, *** *p* < 0.001. **B** Western blot demonstrating that the protein level of POLR2A is downregulated in HFF1 fibroblasts during passages. *n* = 3. * *p* < 0.05, ** p < 0.01, *** *p* < 0.001. **C** HFF1 cells in early passage were treated with 200 μM and 400 μM of H_2_O_2_ for 4 h. SA-β-gal staining was performed at 96 h after H_2_O_2_ treatment. Treatment with H_2_O_2_ significantly enhanced the percentage of SA-β-gal positive cells (the scale bar represents 100 μm). *n* = 3. * *p* < 0.05, ** *p* < 0.01. **D** Western blot revealed reduced POLR2A level in senescent HFF1 cells generated with 200 μM and 400 μM of H_2_O_2_. *n* = 3. * *p* < 0.05, ** *p* < 0.01. **E** The protein level of POLR2A in the brain, heart, lung, and liver organs of young (1 month), middle-aged (9 months), and older (19 months) mice was assessed via western blot. POLR2A was downregulated in the heart, brain, and lung tissues of older mice (19 months), while liver tissue did not exhibit any significant changes. *n* = 3 mice, * *p* < 0.05, ns: no significance.

To evaluate POLR2A expression in cellular senescence, first we employed a replicative senescence cell model with human skin fibroblast HFF1 cells. HFF1 cells at different passages were stained for SA-β-gal, a biomarker of senescence. As the number of cell passages increased, SA-β-gal positive cells gradually enhanced from 13.4% at passage 22 to 42.7% at passage 42 (*p* < 0.001) (Fig. 1A). Similar results were also observed in the replicative senescence model using human lung fibroblast MRC5 cells (Supplementary Fig. 1A). Intriguingly, western blot analysis revealed that POLR2A protein level was decreased in late passages of both HFF1 and MRC5 cells (Fig. 1B and Supplementary Fig. 1B).

Moreover, in the oxidative stress-induced senescence model, treatment of HFF1 cells with H_2_O_2_ at 200 or 400 μM led to much higher percentage of cells positive for SA-β-gal (Figs. 1C, 32.8% and 42.1% compared to 14.6% in the control, *p* < 0.05 and *p* < 0.001 individually) and substantially lower level of POLR2A (Fig. 1D). Similar results were also obtained in H_2_O_2_-treated MRC5 cells. (Supplementary Fig. 1C, D).

In addition, the levels of POLR2A protein in different tissues of C57BL/6 mice with different ages (1, 9 and 19 months) were assayed. POLR2A protein level was reduced in the brain, heart and lung at 9 months as compared to 1 month, which was further decreased in old mice at 19 months (Fig. 1E, *p* < 0.05). Whereas in the liver, only moderate decrease of POLR2A was observed at 9 or 19 months (Fig. 1E). Collectively, these results suggested that the POLR2A protein was downregulated in cellular senescence.

### POLR2A depletion induces cell senescence via activation of p53/p21

**Fig. 2:**
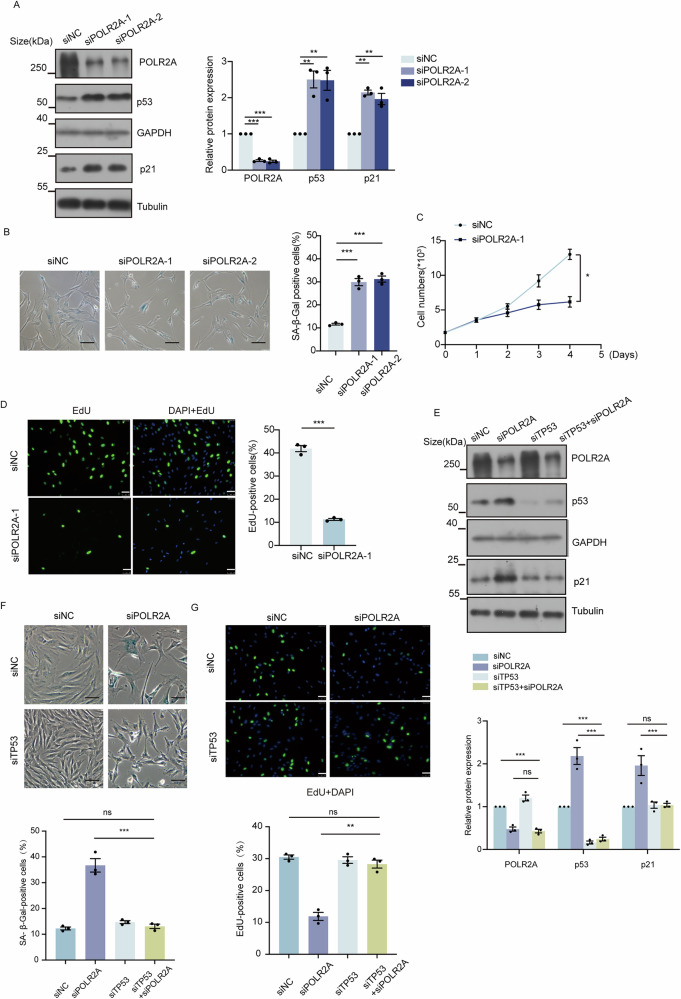
POLR2A downregulation drives cellular senescence through p53/p21 activation. **A** The protein levels of POLR2A, p53, and p21 were analyzed using western blot in the HFF1 cells treated with the siNC or siPOLR2A. *n* = 3. ** *p* < 0.01, *** *p* < 0.001. **B** Representative images of SA-β-gal staining in HFF1 fibroblasts transfected with siPOLR2A or siNC, SA-β-gal staining was performed at 96 h after transfection (the scale bar represents 100 μm). *n* = 3. *** *p* < 0.001. **C** Growth curves of siNC and siPOLR2A-treated HFF1 cells. siRNA-mediated silencing of POLR2A in HFF1 cells inhibited cell proliferation. *n* = 3. * *p* < 0.05. **D** Cell proliferation as measured by EdU incorporation in siNC and siPOLR2A-treated HFF1 cells (the scale bar represents 50 μm). *n* = 3. *** *p* < 0.001. **E** Western blots were used to detect the protein levels of POLR2A, p53, and p21 in HFF1 cells with TP53 and POLR2A knockdown. *n* = 3. *** *p* < 0.001, ns: no significance. **F** Representative images of SA-β-gal staining in HFF1 fibroblasts transfected with siPOLR2A and siTP53 (the scale bar represents 100 μm). *n* = 3. *** *p* < 0.001, ns: no significance. **G** Cell proliferation as measured by EdU incorporation in siPOLR2A and siTP53-treated HFF1 cells (the scale bar represents 50 μm). *n* = 3. ** *p* < 0.01, ns: no significance.

As POLR2A was downregulated in senescent cells, we explored whether depletion of POLR2A induced cell senescence. Specifically, POLR2A was depleted in HFF1 cells using two independent siRNAs (Fig. 2A). Strikingly, POLR2A depletion resulted in significantly enhanced percentage of cells positive for SA-β-gal (Figs. 2B, 29.9% and 31.2% for two siRNA treatment individually vs 11.6% for control, *p* < 0.001 for both). In addition, depletion of POLR2A using siRNA-1 suppressed cell growth (Fig. 2C, *p* < 0.05) and decreased EdU activity (Figs. 2D, 11.3% EdU positive cells after siRNA treatment vs 41.9% in the control, *p* < 0.001). Moreover, the ELISA results revealed a significant increase in the concentration of two key SASP factors, IL-6 and IL-8, in the culture supernatant of HFF1 cells at 96 h following POLR2A knockdown (Supplementary Fig. 2, *p* < 0.001 for both).

To further understand how POLR2A depletion triggered cell senescence, we assayed the cellular senescence pathway effectors p53/p21. As shown in Fig. 2A, POLR2A depletion resulted in significant upregulation of p53 and p21 at protein level. Consistently, SA-β-gal staining of MRC5 cells after POLR2A depletion revealed a notable increase in senescence-positive cells (Supplementary Fig. 3A, B). Western blot showed increased protein level of p53/p21 (Supplementary Fig. 3A).

More intriguingly, co-depletion of POLR2A and p53 failed to up-regulate the cellular senescence marker p21 (Fig. 2E). Consistent with this observation, SA-β-gal staining revealed a much lower percentage of positive cells in co-depletion as compared to POLR2A depletion (Figs. 2F, 12.6% vs 43.3%, *p* < 0.001), whereas EdU assay on cell proliferation indicated that co-depletion of POLR2A and p53 restored the proliferation back to the level comparable to the control cells (Fig. 2G, 13% for POLR2A depletion, 28.6% for p53 depletion and 27.5% for co-depletion vs 30% in the control). These results together indicated that POLR2A depletion induced cellular senescence via activation of p53/p21.

### POLR2A reduction stabilizes p53 via inhibition of MDM4 at the transcriptional level

**Fig. 3:**
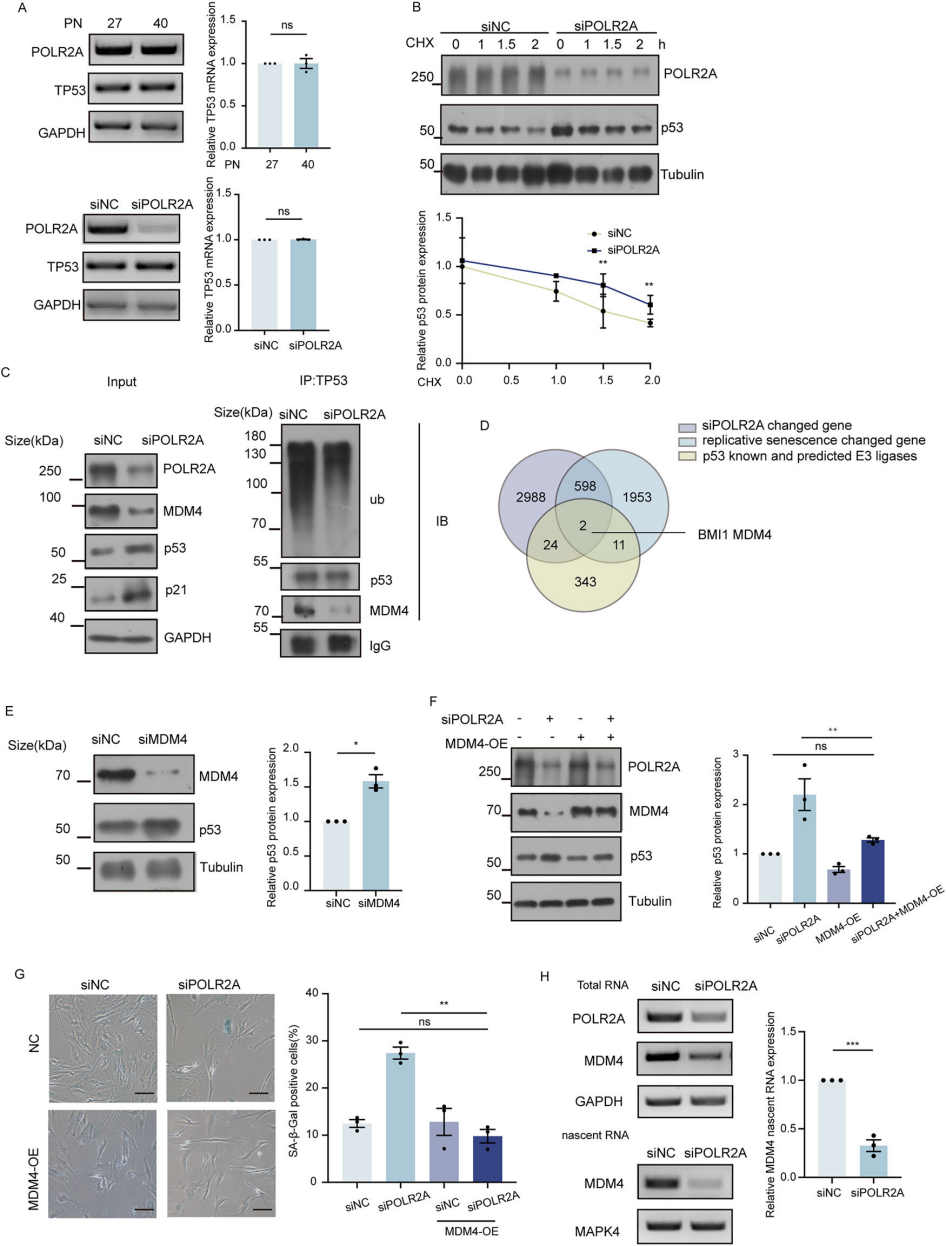
POLR2A depletion stabilizes p53 by transcriptionally inhibiting MDM4 expression. **A** RT-PCR to detect the mRNA level of TP53 in HFF1 fibroblasts after passages or POLR2A depletion. *n* = 3. ns: no significance. **B** After knockdown of POLR2A in HFF1 cells for 60 h, CHX (30 μg/mL) was added to continue treatment for 2 h and samples were collected at 0, 1, 1.5, 2 h for western blot to detect the protein level of p53. *n* = 3. ** *p* < 0.01. **C** Co-IP of endogenous p53 in HFF1 cells with POLR2A knockdown, followed by western blot analysis of ubiquitin and MDM4. **D** Venn diagram illustrating the overlap of differential genes upon replicative senescence, POLR2A knockdown in HFF1, and predicted E3 ligases of p53 based on UbiBrowser 2.0 (http://ubibrowser.bio-it.cn/ubibrowser_v3/home/index). **E** Western blot detecting the protein level of p53 in HFF1 cells with MDM4 knockdown. *n* = 3. * *p* < 0.05. **F** The protein levels of related proteins POLR2A, MDM4 and p53 in HFF1 cells with POLR2A knockdown followed by MDM4 overexpression were detected using western blot. *n* = 3. ** *p* < 0.01, ns: no significance. **G** Representative images of SA-β-gal staining in HFF1 cells with POLR2A knockdown followed by MDM4 overexpression. *n* = 3. ** *p* < 0.01, ns: no significance. **H** Nascent RNA was extracted 72 h after knockdown of POLR2A in HFF1cells and RT-PCR was performed to detect MDM4 nascent RNA level. *n* = 3. *** *p* < 0.001.

To investigate how decreased POLR2A expression in senescent cells induced enhanced expression of p53, we first examined p53 mRNA levels in cell model of replicative senescence as well as in cells with POLR2A depletion. As shown in Fig. 3A, RT-PCR results revealed no increase in p53 mRNA expression, suggesting enhanced expression of p53 occurred post-transcriptionally. Next, POLR2A depleted HFF1 cells were treated with CHX and the stability of p53 protein was assayed. The result implied that POLR2A reduction delayed the degradation of p53 at protein level (Fig. 3B). Furthermore, co-IP of endogenous p53 or ubiquitin in HFF1 cells with POLR2A depletion indicated that ubiquitination of p53 was substantially reduced as compared to control knockdown (Fig. 3C and Supplementary Fig. 4), suggesting that POLR2A reduction enhanced p53 expression by inhibiting the proteasomal degradation of p53 protein.

To investigate the potential E3 ligase mediating p53 stabilization upon POLR2A knockdown, we assayed eight reported p53-associated E3 ligases (MDM2, FBXW7, Pirh2, UBE4B, TRIM24, TRIM28, TRIM69 and TRIM39) for the expression. As shown in Supplementary Fig. 5A, POLR2A depletion in HFF1 cells did not significantly alter the protein levels of any of these eight E3 ligases. Furthermore, Co-IP assay demonstrated that the interaction between p53 and each of these E3 ligases also remained unchanged after POLR2A knockdown (Supplementary Fig. 5B), suggesting the involvement of other factors or mechanism for the observed phenotype.

As POLR2A is the largest subunit of RNA polymerase II which plays a key role in mRNA transcription, we hypothesized that POLR2A reduction induced p53 expression via transcriptional regulation of specific target gene functioning in p53 degradation. To further search for such a gene systematically, genes with significant expression changes in RNA-seq of POLR2A depletion (Supplementary Table S4) and replicative senescence model (Supplementary Table S5) were overlapped with genes encoding E3 ubiquitin ligases correlated to p53 degradation predicted using UbiBrowser 2.0 (Supplementary Table S6), which narrowed down the candidates to two genes: BMI1 and MDM4 (Fig. 3D). However, knockdown BMI1 did not promote p53 express, only knockdown MDM4 led to substantial enhanced level of p53 protein (Fig. 3E). Co-IP assay further revealed that the binding of MDM4 to p53 protein was significantly decreased after POLR2A depletion (Fig. 3C). Overexpression of MDM4 in HFF1 cells followed by POLR2A depletion did not result in enhanced level of p53 protein (Fig. 3F). In addition, the increase of SA-β-gal positive cells induced by POLR2A depletion was reversed after overexpression of MDM4 (Figs. 3G, 9.3% vs 27.4%, *p* < 0.01). Finally, chromatin-associated nascent RNA was assayed in HFF1 cells after POLR2A depletion, as shown in Fig. 3H, RT-PCR results indicated a striking decrease of nascent MDM4 RNA, confirming that POLR2A depletion led to a transcriptional inhibition on MDM4. Collectively, our data supported that POLR2A reduction stabilized p53 protein via inhibition on MDM4 at the transcriptional level.

### LMO7 promotes POLR2A ubiquitination in cellular senescence

**Fig. 4:**
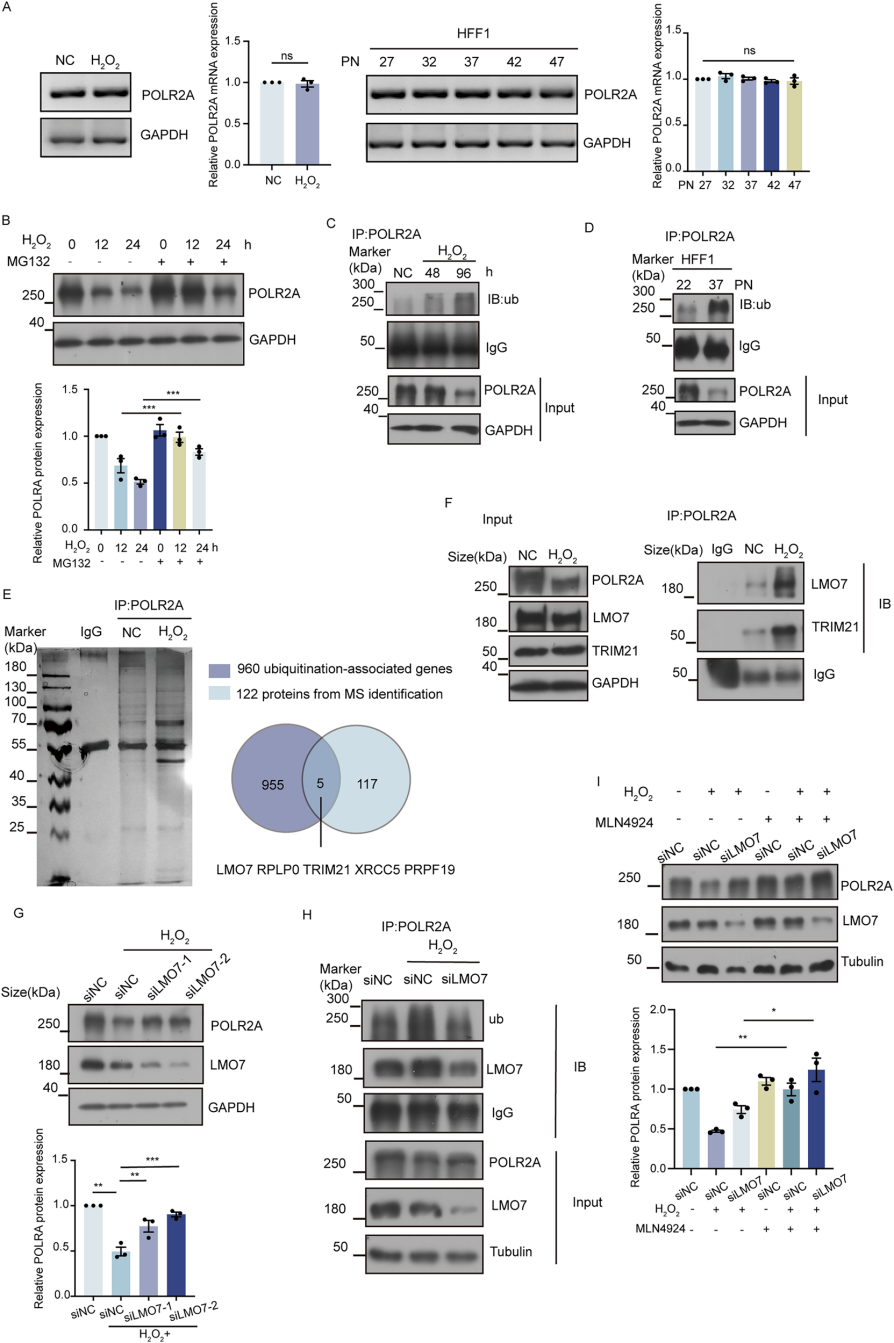
POLR2A is degraded by LMO7-mediated ubiquitination during cellular senescence. **A** RT-PCR to detect the mRNA level of POLR2A in H_2_O_2_ (200 μM)-induced senescent and replicative senescent HFF1 cells. *n* = 3. ns: no significance. **B** After H_2_O_2_ (200 μM) treatment of HFF1 cells for 72 h, MG132 (20 μg/mL) was added to continue treatment for 24 h and samples were collected at 0, 12, and 24 h for western blot to detect the protein level of POLR2A. *n* = 3. *** *p* < 0.001. **C** Co-IP of endogenous POLR2A in H_2_O_2_ (200 μM)-treated HFF1 cells, followed by western blot analysis of ubiquitin. MG132 (20 μg/mL) was added 2 h before harvest. **D** Co-IP of endogenous POLR2A in HFF1 cells after passages, followed by western blot analysis of ubiquitin. **E** Silver staining showing the IP of endogenous POLR2A in H_2_O_2_ (200 μM)-treated HFF1 cells. Venn diagram illustrating the overlap of protein candidates in MS analyses and 960 ubiquitination-associated genes included in the Human Ubiquitin siRNA library. **F** Differentially bound proteins in MS results were validated by western blot. **G** HFF1 cells were treated with H_2_O_2_ (200 μM) for 4 h, and then transfected with siNC and siLMO7 within 24 h. Samples were collected at 72 h post-transfection for the western blot detection of POLR2A and LMO7. *n* = 3. ** *p* < 0.01, *** *p* < 0.001. **H** HFF1 cells were treated with H_2_O_2_ (200 μM) for 4 h, and then transfected with siNC and siLMO7 within 24 h. Western blot analysis of POLR2A ubiquitination at 72 h post-transfection. MG132 (20 μg/mL) was added 2 h before harvest. **I** HFF1 cells were treated with H_2_O_2_ (200 μM) for 4 h, and then transfected with siNC and siLMO7 within 24 h. MLN4924 (500 ng/mL) was added 48 h post-transfection. Samples were collected at 72 h for Western blot analysis of POLR2A and LMO7 protein levels. *n* = 3. * *p* < 0.05, ** *p* < 0.01.

To explore the mechanism of POLR2A downregulation in senescence, we first examined whether POLR2A was transcriptionally downregulated at the mRNA level in replicative senescence and H_2_O_2_-induced senescence cell models. The RT-PCR results showed that the level of POLR2A mRNA did not change upon H_2_O_2_ treatment or among different cell passages (Fig. 4A), suggesting POLR2A reduction in senescence was not due to impaired transcription. Next, we focused on the potential mechanisms of POLR2A protein degradation. Treatment of HFF1 cells with H_2_O_2_ followed by the proteasome inhibitor MG132 significantly restored POLR2A protein level in senescent cells (Fig. 4B). Consistently, further analysis of co-IP assays revealed significant enhanced protein ubiquitination of POLR2A in both senescent cell models as shown in Fig. 4C, D, indicating that POLR2A reduction in senescence was due to proteasome-mediated protein degradation.

In an attempt to identify key factors involved in POLR2A degradation by ubiquitination during cell senescence, protein IP was performed on H_2_O_2_-induced senescent cells followed by MS analysis, which revealed 122 protein candidates with significantly enhanced interaction to POLR2A in senescent cells (Fig. 4E and Supplementary Table S7). Overlap of these proteins with 960 ubiquitination-associated genes included in the Human Ubiquitin siRNA library [30] revealed five candidates: LMO7, RPLP0, TRIM21, XRCC5 and PRPF19. Among them, LMO7 has been reported to mediate protein ubiquitination degradation [31], and TRIM21 is a known E3 ubiquitin ligase. Intriguingly, western blot suggested that the binding of LMO7 and TRIM21 to POLR2A was increased following H_2_O_2_ treatment (Fig. 4F). Further depletion of LMO7 or TRIM21 in H_2_O_2_-induced senescent cells indicated that knockdown of LMO7 significantly inhibited POLR2A reduction whereas knockdown of TRIM21 showed no obvious effect (Fig. 4G and Supplementary Fig. 6). Consistent with this finding, co-IP assays demonstrated that the ubiquitination of POLR2A in senescent cells was reduced substantially upon LMO7 knockdown (Fig. 4H). Moreover, POLR2A protein level was dramatically restored when H_2_O_2_-induced senescent cells with or without LMO7 depletion were treated with the ubiquitination inhibitor MLN4924 (Fig. 4I). Together, these results pinpointed a vital role of LMO7 in POLR2A degradation during cell senescence.

### Activation of endogenous POLR2A expression alleviates cell senescence

**Fig. 5:**
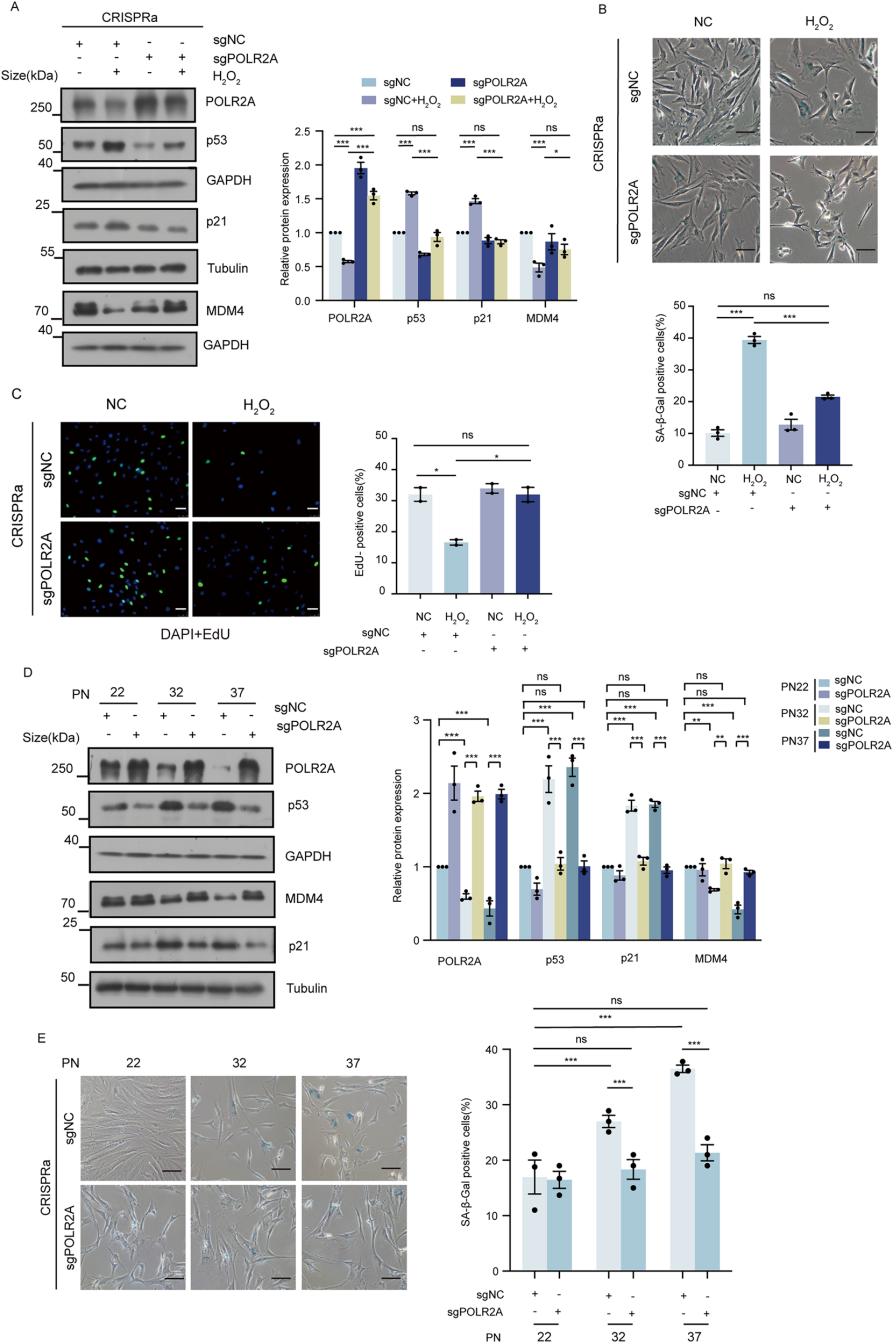
Activation of endogenous POLR2A reverses cellular senescence. **A** HFF1 cells was treated with CRISPRa-mediated POLR2A overexpression, and samples were collected at 96 h following a 4-h treatment with H_2_O_2_ (200 μM). Western blot was conducted to ascertain the relative protein levels of POLR2A, p53, p21 and MDM4. *n* = 3. * *p* < 0.05, *** *p* < 0.001, ns: no significance. (B) HFF1 cells was treated with CRISPRa-mediated POLR2A overexpression, and SA-β-gal staining was performed at 96 h following a 4-h treatment with H_2_O_2_ (200 μM). *n* = 3. *** *p* < 0.001, ns: no significance. **C** HFF1 cells was treated with CRISPRa-mediated POLR2A overexpression, and cell proliferation by EdU staining was measured at 96 h following a 4-h treatment with H_2_O_2_ (200 μM). *n* = 3. ** *p* < 0.01, *** *p* < 0.001, ns: no significance. **D** Cell passaging was performed after CRISPRa-mediated POLR2A activation in HFF1 cells. The protein levels of the associated proteins POLR2A, p53, p21 and MDM4 were detected by western blot. n = 3. ** *p* < 0.01, *** *p* < 0.001, ns: no significance. **E** Representative images of SA-β-gal staining in HFF1 cells with cell passaging followed by CRISPRa-mediated POLR2A activation. *n* = 3. *** *p* < 0.001, ns: no significance.

To further investigate the correlation between POLR2A expression level and cellular senescence, we activated endogenous POLR2A expression using CRISPRa technique in HFF1 cells. The effective activation of POLR2A was verified by western blot (Fig. 5A). Activation of endogenous POLR2A expression led to significant decrease of SA-β-gal positive cells (39.4% vs 21.5%, *p* < 0.001) after HFF1 cells were treated with H_2_O_2_ (Fig. 5B) as well as an increase in cell proliferation (Fig. 5C, EdU positive cells of 16.6% vs 31.9%, *p* < 0.05). In addition, H_2_O_2_ treatment of HFF1 cells resulted in decreased level of MDM4 and enhanced level of p53/p21, whereas the same treatment of cells after CRISPRa of POLR2A led to higher level of MDM4 and lower level of p53/p21 (Fig. 5A).

Next, CRISPRa of POLR2A was performed in replicative senescent cell model, western blot assay revealed that POLR2A expression was activated and maintained at similar levels in the three passages of HFF1 cells (Fig. 5D). Intriguingly, while staining of the control cells indicated significant increased SA-β-gal activity in senescent cells (Figs. 5E, 27.1% and 36.5% for PN32 and 37 vs 16.9% for PN22, *p* < 0.001), staining of the cells with CRISPRa of POLR2A at different passages revealed only a mild increase of SA-β-gal activity without statistical significance (Figs. 5E, 18.3% and 21.3% for PN32 and 37 vs 16.9% for PN22). Consistently, western blot assay revealed low levels of p53/p21 and high levels of MDM4 in HFF1 cells with CRISPRa of POLR2A of high passage number (Fig. 5D). Overall, our results supported that POLR2A was a potential suppressor of cellular senescence, and such effect was achieved via its regulation on p53/p21 level mediated by MDM4.

## Discussion

In recent years, several articles have reported that POLR2A is closely associated with senescence [14, 16, 17], however, the exact relationship between POLR2A and cellular senescence has not been fully elucidated. Owing to the importance of the POLR2A protein, the mechanism of its degradation has also received widespread attention. In this study, we reported that POLR2A served as a critical guardian against cellular senescence, and the LMO7-induced ubiquitination and degradation of POLR2A protein drived cellular senescence through the MDM4/p53/p21 axis.

Our previous studies revealed that POLR2A mediated cellular senescence caused by knockdown of a multifunctional gene XAB2 [17]. Downregulating POLR2A alone induced cellular senescence via p53/p21 activation, while its re-expression rescued senescence in XAB2-deficient cells [17]. These findings suggested POLR2A might serve as a potential regulator of cellular senescence. Here, we systematically investigated the function and underlying mechanism of POLR2A in cellular senescence. We first established two models of cellular senescence induced by replication stress and oxidative stress in normal human fibroblasts and validated that POLR2A protein level was indeed downregulated in senescent cells. Besides, we observed significant downregulation of POLR2A protein in the brain, heart, and lung of aged mouse, supporting its role in cellular senescence in vivo. This was consistent with previous reports that POLR2A expression was significantly downregulated in Werner syndrome patients or normal old donor cells, and in aged human tissues, including aorta, spleen, and adrenal gland [14, 16]. Following POLR2A knockdown in HFF1 and MRC5 cells, we observed a significant increase in senescence phenotypes, such as elevated SA-β-gal activity and reduced cell proliferation.

Furthermore, the p53/p21 senescence pathway was significantly activated following POLR2A knockdown, and co-depletion of POLR2A and p53 rescued the senescence phenotype, confirming that POLR2A-induced senescence was p53-dependent. However, the mechanism by which POLR2A downregulation leads to increased p53 protein level in normal fibroblasts remains unclear, which sparked our strong research interest. First, we detected p53 mRNA level in POLR2A-knockdown-induced senescent cells via RT-PCR and validated it using a replicative senescence model. Results revealed no significant change in p53 mRNA expression in either model. Consequently, we hypothesized that POLR2A regulated p53 at the post-transcriptional level. Next, endogenous co-IP experiments demonstrated that p53 protein ubiquitination was significantly reduced after POLR2A knockdown. This suggested that POLR2A downregulation affected the ubiquitination and degradation process of the p53 protein. However, we found no evidence that p53 stabilization upon POLR2A knockdown was mediated by the tested subset of eight reported p53-associated E3 ligases, including MDM2, FBXW7, Pirh2, UBE4B, TRIM24, TRIM28, TRIM69 and TRIM39[19–27]. Given POLR2A’s key role in gene transcription, we further performed RNA-seq analysis on RNA extracted from POLR2A-knockdown cells, alongside RNA from replicative senescent cells as a control. Among the altered genes, we ultimately identified MDM4, a well-known p53 negative regulator, as being substantially downregulated in both types of senescent cells. Co-IP assay further demonstrated that the binding of MDM4 to p53 protein was significantly decreased after POLR2A depletion. As a family member of E3 ligase MDM2, MDM4 lacks E3 ligase activity but functions as a critical cellular activator. It can heterodimerizes with MDM2 to convert it from a monoubiquitinating into a polyubiquitinating E3 ligase toward p53, thereby enhancing p53 degradation [32]. Therefore, we hypothesize that POLR2A knockdown downregulates MDM4, which in turn reduces the binding of the MDM2-MDM4 heterodimer to p53, thus promoting p53 stability. MDM4 can also inhibit the transactivation activity of p53 by binding to its transactivation domain [33]. Reduction of MDM4 or the alternative splicing transition from the MDM4-FL to MDM4-S has been reported to induce cellular senescence [34]. In POLR2A-deficient cells, restoring MDM4 expression rescued the elevated p53 and SA-β-gal activity, establishing MDM4 as a key mediator of p53 upregulation following POLR2A knockdown. In addition, the nascent MDM4 RNA was strikingly decreased after POLR2A depletion. Collectively, these evidences demonstrated that POLR2A reduction stabilized p53 protein by transcriptionally inhibiting MDM4 expression.

Given the functional significance of POLR2A downregulation and its observed decrease in senescent cells, we sought to investigate the specific mechanism behind this reduction during cellular senescence. We first examined POLR2A mRNA levels in H_2_O_2_-induced senescent cells and replicative senescence cells, the results showed no significant changes in mRNA expression. Moreover, the proteasome inhibitor MG132 significantly preserved POLR2A protein level and co-IP experiments in H_2_O_2_-induced cells confirmed elevated POLR2A protein ubiquitination levels. Based on these findings, we concluded that POLR2A underwent accelerated ubiquitination and degradation in senescent cells. Then, we performed MS analysis on the IP samples. The results revealed five ubiquitin-related proteins with increased binding to POLR2A during H_2_O_2_-induced cellular senescence, among which LMO7 showed the most pronounced increase. It has been reported that E3 ubiquitin ligases, such as Nedd4, WWP2, CRL4^CSA^, ARMC5/CUL3/RBX1, function in POLR2A ubiquitination under DNA damage or normal condition [7–10]. Importantly, our results showed that depletion of LMO7 significantly inhibited the ubiquitination of POLR2A and POLR2A reduction in H_2_O_2_-induced senescent cells, pinpointing LMO7’s vital role in POLR2A degradation during cell senescence. LIM domain protein 7 (LMO7) is a large protein that coordinates multiple protein-protein interactions. Its structure contains multiple LIM domains (a unique cysteine-rich zinc-binding domain), which serve as protein-protein interaction motifs involved in cell growth, differentiation, and cytoskeletal organization [35]. Additionally, the LMO7 protein contains a calmodulin-homologous domain crucial for regulating cell morphology and signaling, a PDZ domain, and an F-box domain capable of participating in ubiquitination [36]. Among these, the function of LMO7 as an E3 ubiquitin ligase has attracted widespread attention. Recent studies indicated that LMO7 directly bound to Foxp1 through its LIM domain, promoting Foxp1’s ubiquitination and degradation, which led to immune evasion in pancreatic ductal adenocarcinoma [37]. Overexpression of LMO7 significantly alleviated hepatic steatosis, inflammation, and fibrosis by promoting the K48-linked ubiquitination and degradation of TRIM47 [31].

To definitively establish POLR2A’s role as a guardian against senescence, we asked whether its re-expression could mediate functional rescue of the senescence phenotype. Using CRISPRa technology [38], we first activated endogenous POLR2A expression in HFF1 cells, followed by H_2_O_2_-induced senescence. We observed that activating POLR2A expression in H₂O₂-induced senescent cells downregulated the SA-β-gal activity and rescued cell proliferation. Concomitantly, it inactivated the p53/p21 pathway and restored MDM4 expression. These findings were recapitulated in the replicative senescence model.

**Fig. 6:**
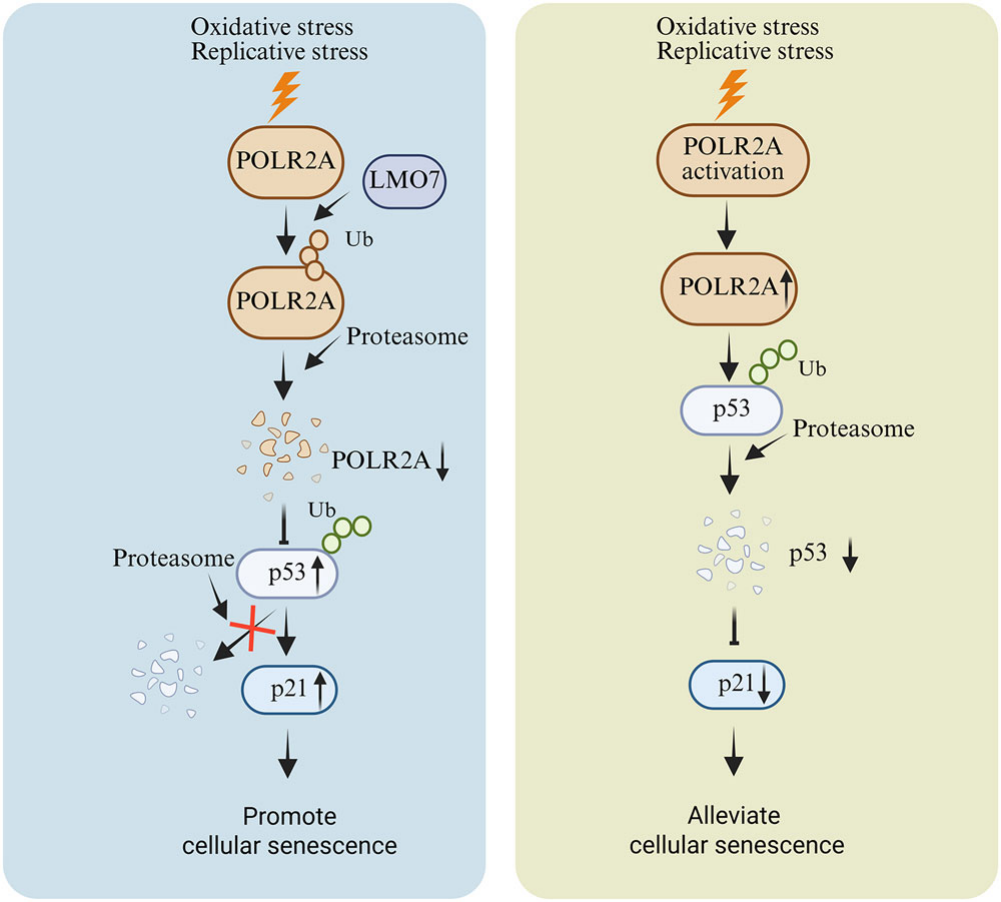
Model for POLR2A degradation and function in cellular senescence.

Based on the above data, we propose a model for the role of POLR2A in cellular senescence. As illustrated in Fig. 6, under external stressors such as oxidative stress or telomere damage, the POLR2A protein within the cell undergoes degradation via the ubiquitin-proteasome pathway mediated by E3 ubiquitin ligase LMO7. The subsequent downregulation of POLR2A impairs MDM4 transcription, thereby relieving its inhibition of p53. This leads to activation of the p53/p21 pathway and ultimately induces cellular senescence. Restoring POLR2A expression in cells undergoing premature aging induced by external stress can effectively counteract cellular senescence.

Although our study provides deep insights into the regulatory role of POLR2A in cellular senescence, it also has certain limitations that warrant further investigation. The regulatory function of POLR2A in cellular senescence should be examined in other senescence models in vitro and in vivo. A more comprehensive understanding of the molecular details underlying LMO7-mediated ubiquitination of POLR2A during senescence awaits further study.

## Supplementary information

clean version of Supplementary Figure legends

Supplemental-Figure 1

Supplemental-Figure 2

Supplemental-Figure 3

Supplemental-Figure 4

Supplemental-Figure 5

Supplemental-Figure 6

Table S1

Table S2

Table S3

Table S4

Table S5

Table S6

Table S7

Original Data

## Data Availability

The data that support this study are available from the corresponding author upon request. The raw sequence data reported in this paper have been deposited in the Genome Sequence Archive in National Genomics Data Center[39], China National Center for Bioinformation / Beijing Institute of Genomics, Chinese Academy of Sciences (GSA-Human: HRA013617) that are publicly accessible at https://ngdc.cncb.ac.cn/gsa-human/browse/HRA013617.
