# Supplementary material for: LMO7-mediated POLR2A degradation promotes cellular senescence through the MDM4/p53/p21 axis
Source: Cell Death Dis. 2026 Mar 28;17(1):421. doi: 10.1038/s41419-026-08679-0 (PMC13149549; doi:10.1038/s41419-026-08679-0)
Supplement: Supplementary file 1 — clean version of Supplementary Figure legends [file 41419_2026_8679_MOESM1_ESM.docx]

**Supplementary Figure legends:**

**Supplementary Fig. 1 POLR2A expression is reduced in senescent MRC5 cells**

(A) Representative images of SA-β-gal staining at different passage number (PN) of 22, 32, 37, 42, and 47 in MRC5 cells (the scale bar represents 100 μm). The percentage of SA-β-gal positive (blue) cells had significant increase in late passage (PN 32, 37, 42). n = 3. ** p < 0.01, *** p < 0.001.

(B) Western blot demonstrating that the protein level of POLR2A is downregulated in MRC5 fibroblasts during passages. n = 3. ** p < 0.01, *** p < 0.001.

(C) MRC5 cells in early passage were treated with 150 μM and 300 μM of H_2_O_2_ for 4 hours. SA-β-gal staining was performed at 96 hours after H_2_O_2_ treatment. Treatment with H_2_O_2_ significantly enhanced the percentage of SA-β-gal positive cells (the scale bar represents 100 μm). n = 3. ** p < 0.01.

(D) Western blot revealed reduced POLR2A level in senescent HFF1 cells generated with 150 μM and 300 μM of H_2_O_2_. n = 3. ** p < 0.01.

**Supplementary Fig. 2** Knockdown of POLR2A leads to increased SASP secretion in HFF1 cells.

ELISA analysis of IL-6 and IL-8 secretion from POLR2A knockdown HFF1 cells. n = 3. *** p < 0.001.

**Supplementary Fig. 3 POLR2A downregulation drives cellular senescence through p53/p21 activation in MRC5 cells**

A) The protein levels of POLR2A, p53, and p21 were analyzed using western blot in the MRC5 cells treated with the siNC or siPOLR2A. n = 3. *** p < 0.001.

(B) Representative images of SA-β-gal staining in MRC5 fibroblasts transfected with siPOLR2A or siNC, SA-β-gal staining was performed at 96 hours after transfection (the scale bar represents 100 μm). n = 3. *** p < 0.001.

**Supplementary Fig. 4** Downregulation of POLR2A reduces the ubiquitination of p53

Co-IP of endogenous ubiquitin in HFF1 cells with POLR2A knockdown, followed by western blot analysis of p53.

**Supplementary Fig. 5 POLR2A depletion stabilizes p53 independently of its known E3 ligases**

(A) Western blot detecting the protein levels of MDM2, FBXW7, Pirh2, UBE4B, TRIM24, TRIM28, TRIM69 and TRIM39 in HFF1 cells with POLR2A knockdown. n = 3. ns: no significance.

(B) Co-IP of endogenous p53 in HFF1 cells with POLR2A knockdown, followed by western blot analysis of MDM2, FBXW7, Pirh2, UBE4B, TRIM24, TRIM28, TRIM69 and TRIM39.

**Supplementary Fig. 6 POLR2A degradation during cellular senescence is independent of TRIM21**

HFF1 cells were treated with H_2_O_2_ (200 μM) for 4 hours and then transfected with siNC and siTRIM21 within 24 hours. Samples were collected at 72 hours post-transfection for the western blot detection of POLR2A and TRIM21. n =3. *** p < 0.001. ns: no significance.
