# Supplementary figures and images for: LMO7-mediated POLR2A degradation promotes cellular senescence through the MDM4/p53/p21 axis
Source: Cell Death Dis. 2026 Mar 28;17(1):421. doi: 10.1038/s41419-026-08679-0 (PMC13149549; doi:10.1038/s41419-026-08679-0)

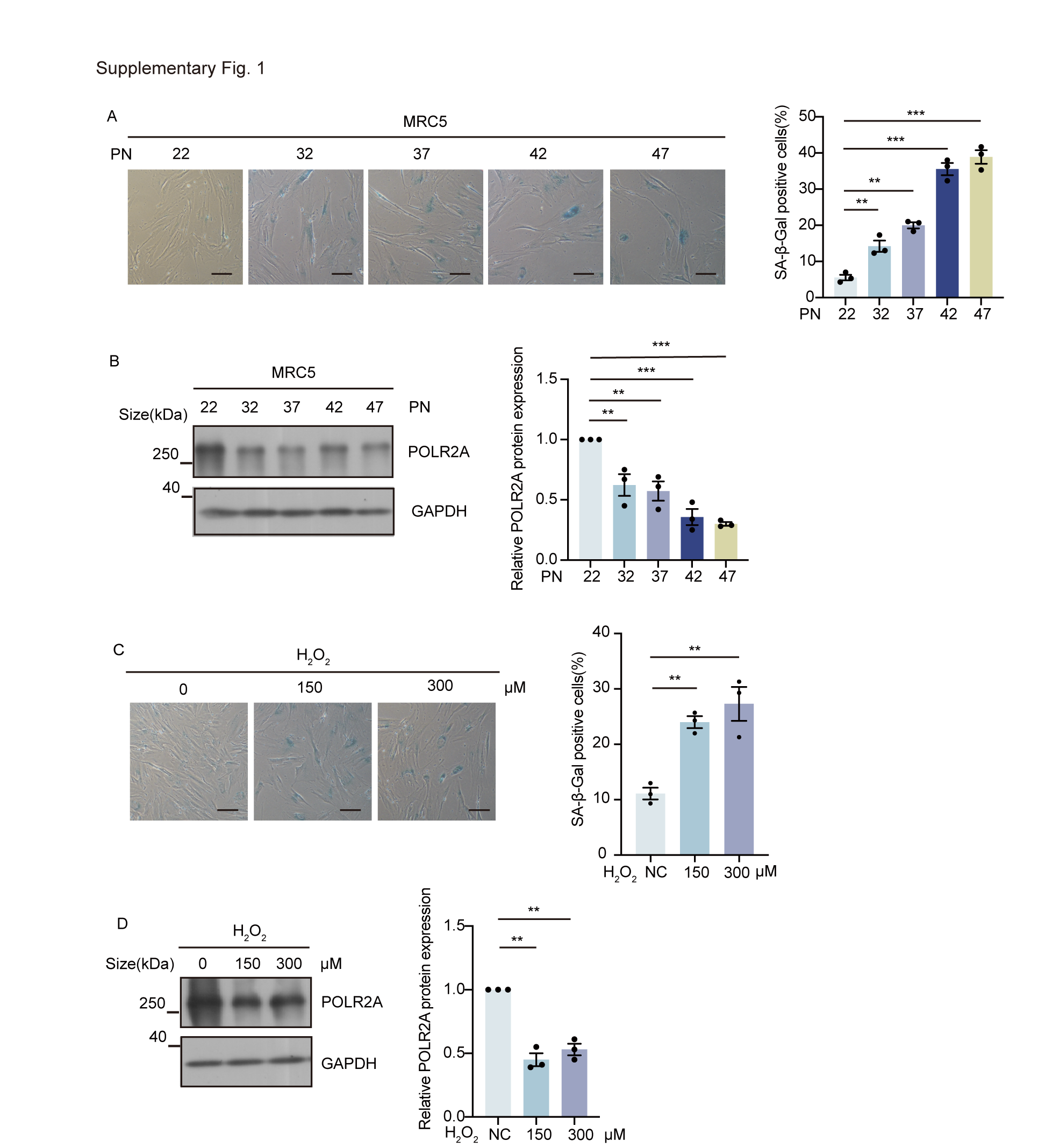

Supplement: Supplementary file 2 — Supplemental-Figure 1 [file 41419_2026_8679_MOESM2_ESM.tif]

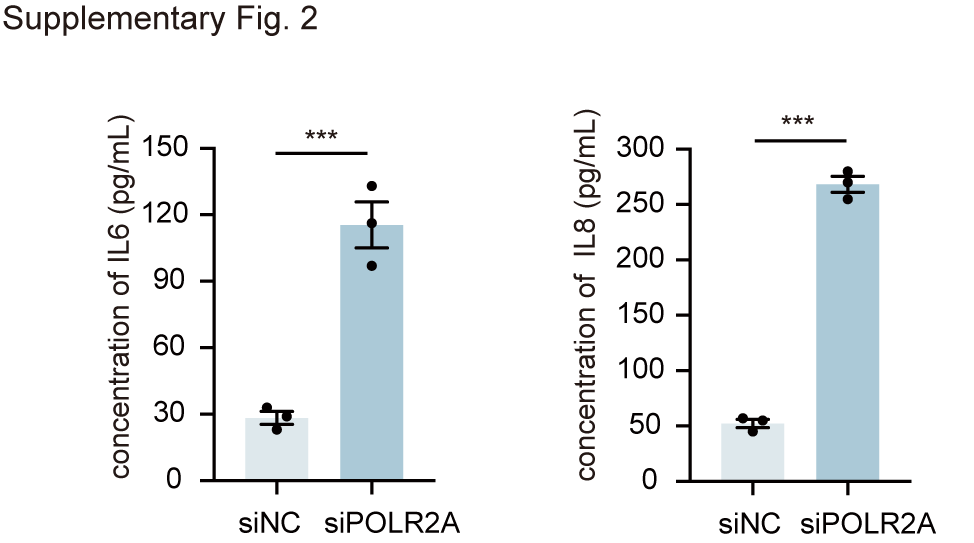

Supplement: Supplementary file 3 — Supplemental-Figure 2 [file 41419_2026_8679_MOESM3_ESM.tif]

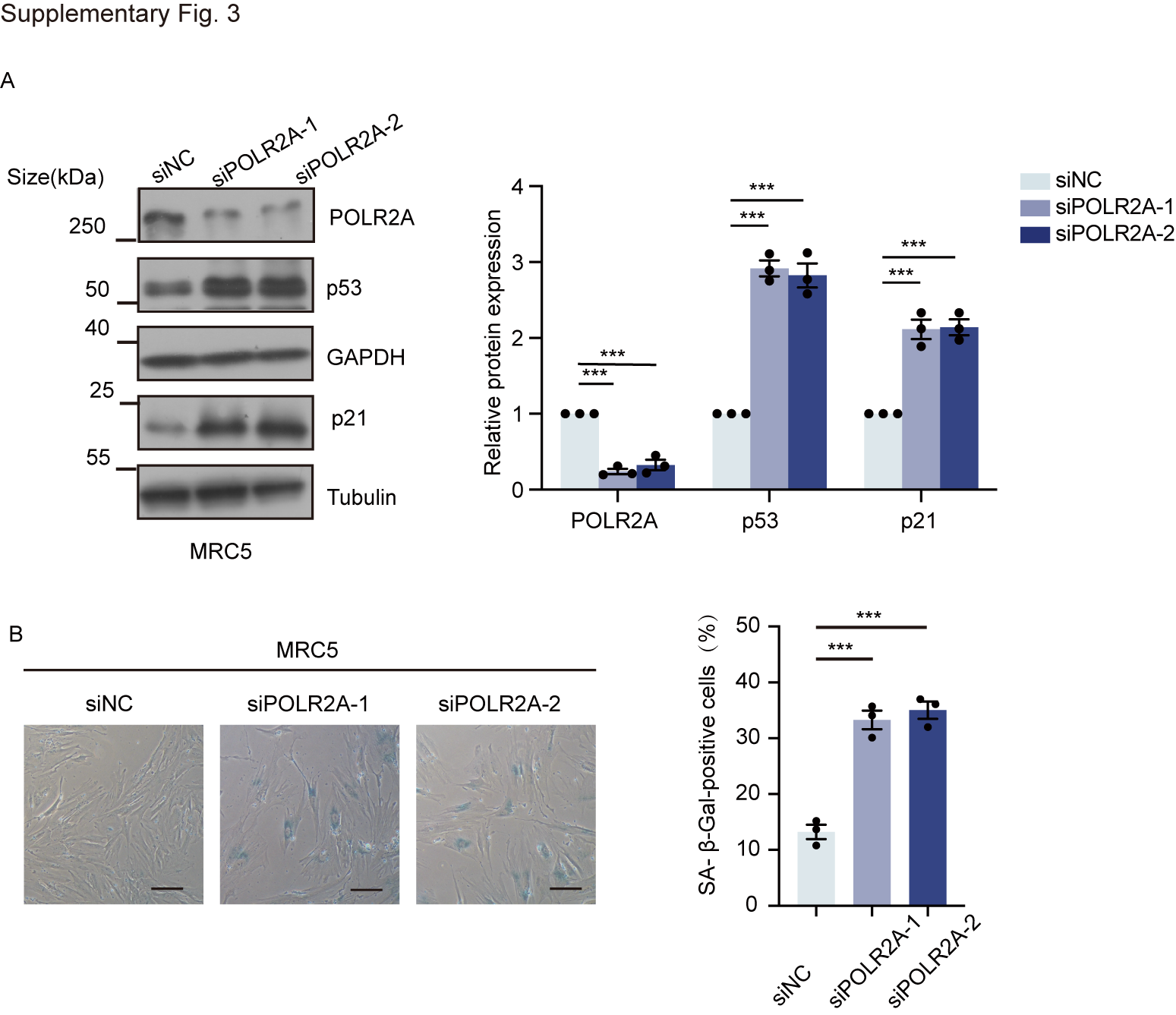

Supplement: Supplementary file 4 — Supplemental-Figure 3 [file 41419_2026_8679_MOESM4_ESM.tif]

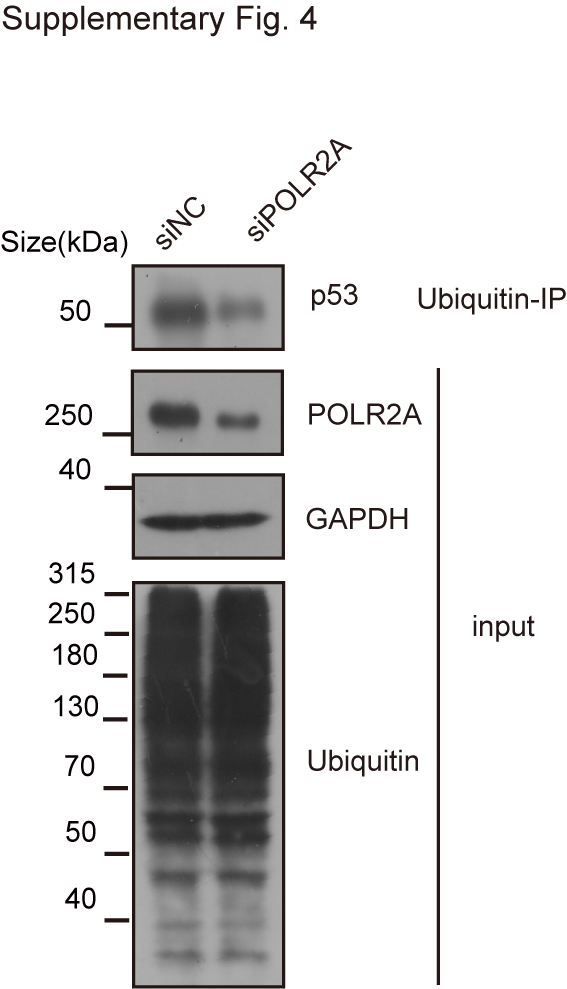

Supplement: Supplementary file 5 — Supplemental-Figure 4 [file 41419_2026_8679_MOESM5_ESM.tif]

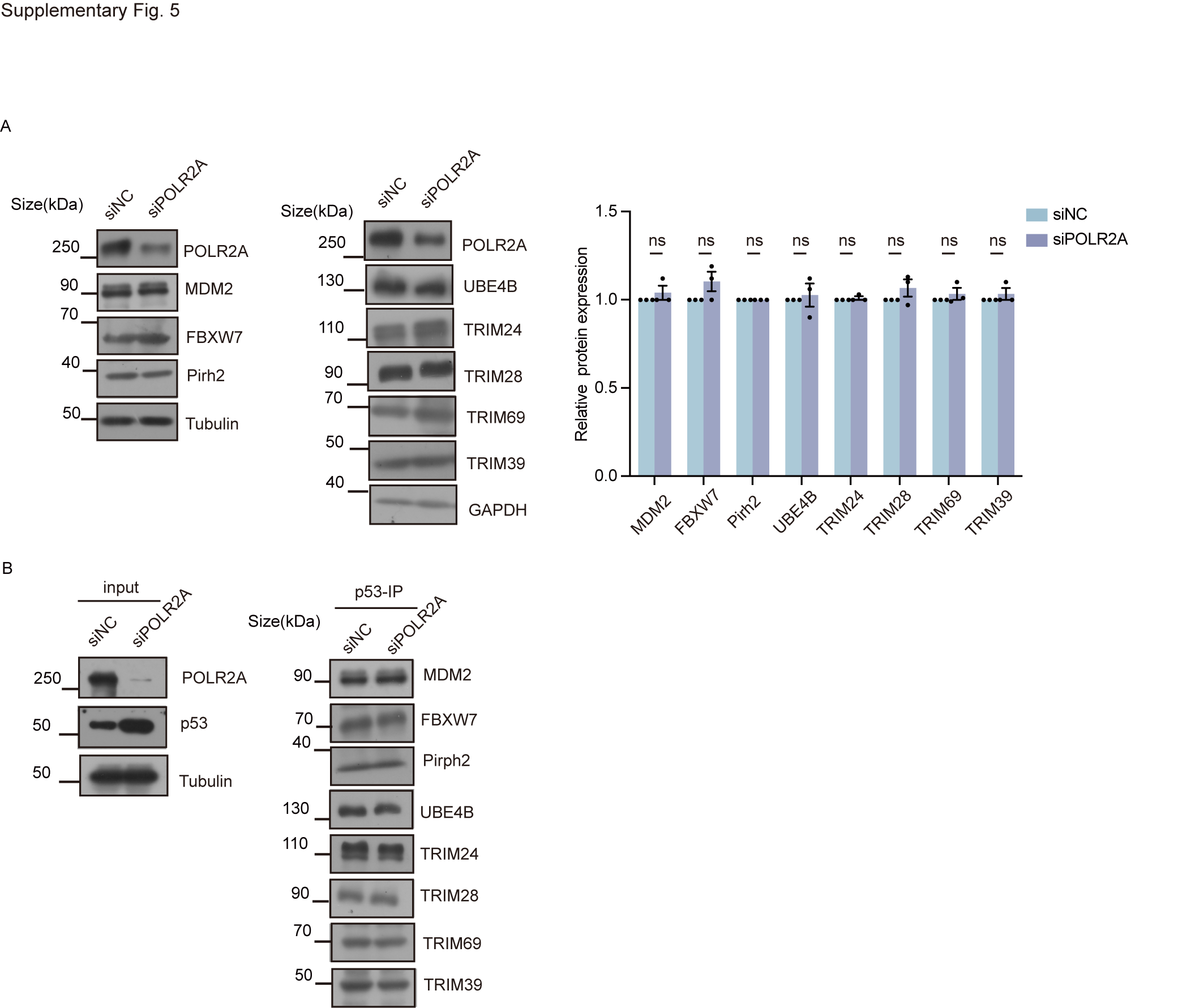

Supplement: Supplementary file 6 — Supplemental-Figure 5 [file 41419_2026_8679_MOESM6_ESM.tif]

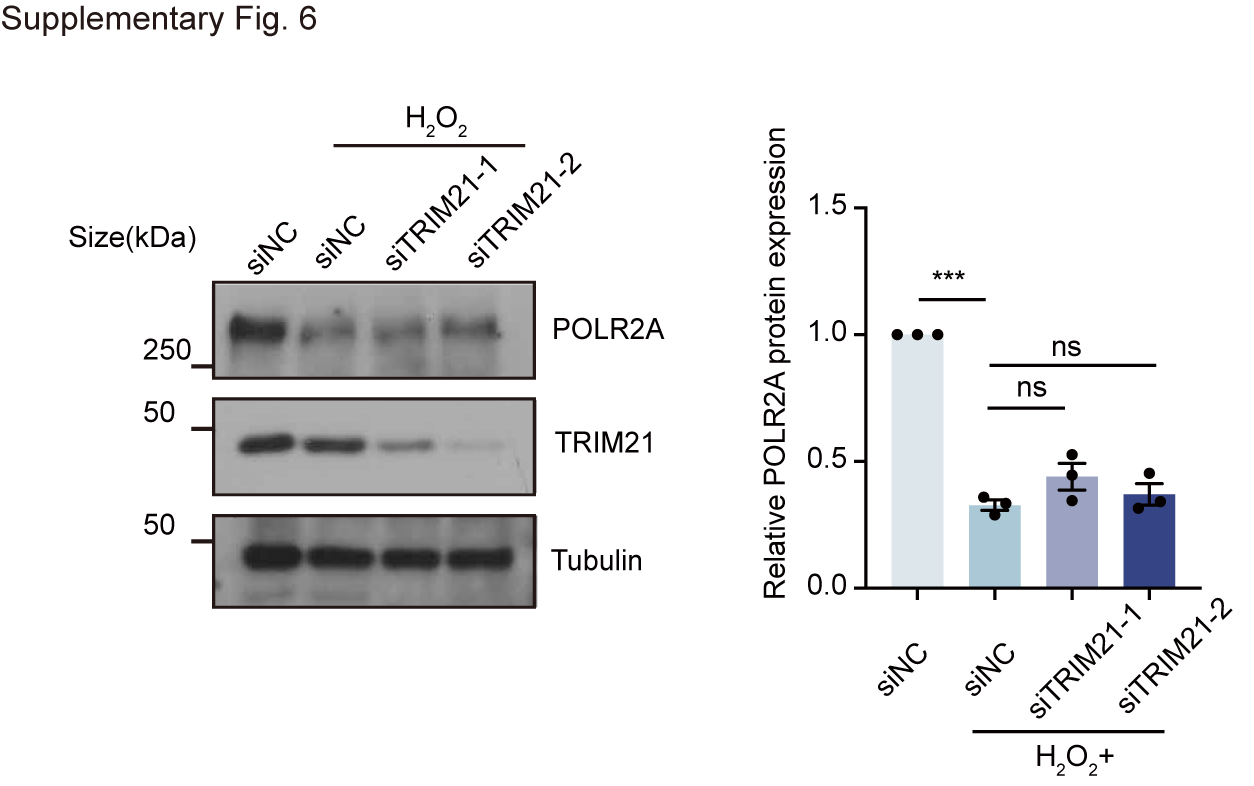

Supplement: Supplementary file 7 — Supplemental-Figure 6 [file 41419_2026_8679_MOESM7_ESM.tif]
