# Supplementary material for: LMO7-mediated POLR2A degradation promotes cellular senescence through the MDM4/p53/p21 axis
Source: Cell Death Dis. 2026 Mar 28;17(1):421. doi: 10.1038/s41419-026-08679-0 (PMC13149549; doi:10.1038/s41419-026-08679-0)

Figure1

B

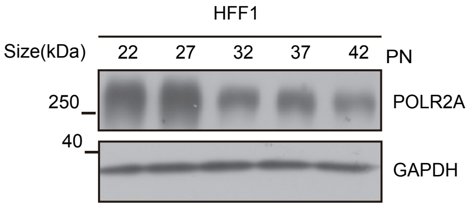


B

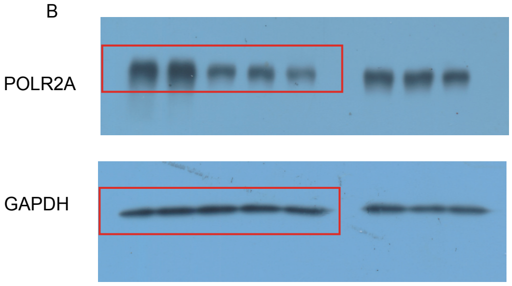


D

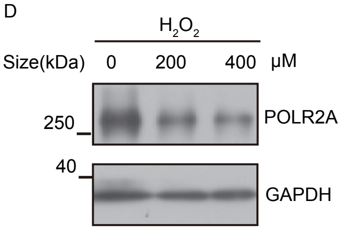


D

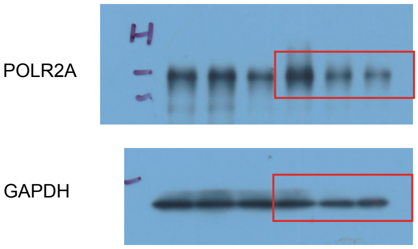


E

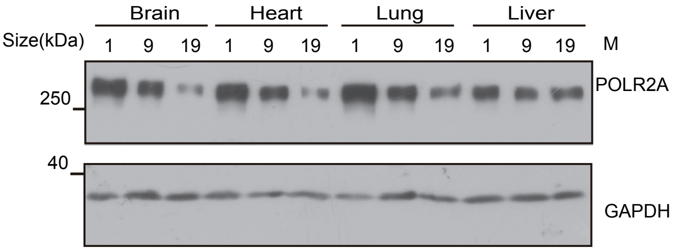


E

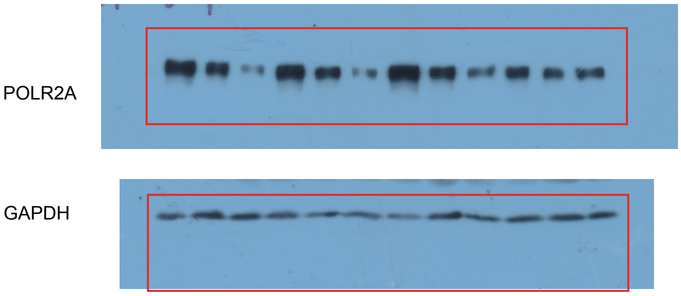

# Figure2

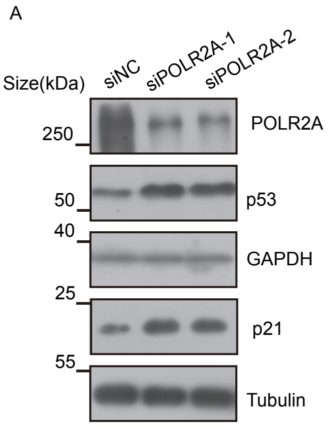
A
Size(kDa)
siNC
siPOLR2A-1
siPOLR2A-2
250
POLR2A
50
p53
40
GAPDH
25
p21
55
Tubulin

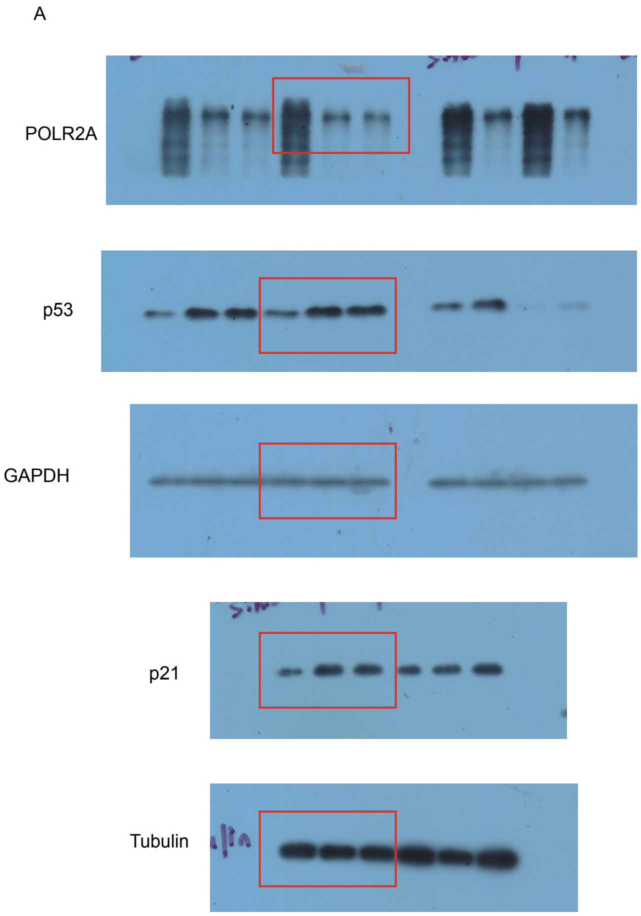
A
POLR2A
p53
GAPDH
p21
Tubulin

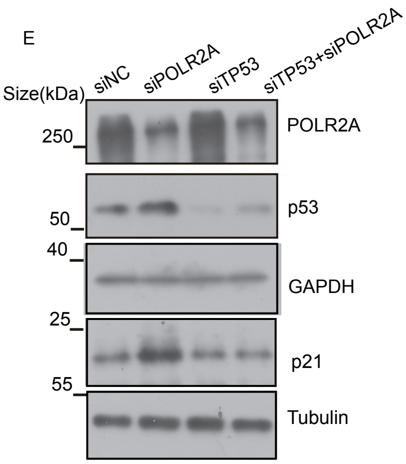
E
Size(kDa)
siNC
siPOLR2A
siTP53
siTP53+siPOLR2A
250
POLR2A
50
p53
40
GAPDH
25
p21
55
Tubulin

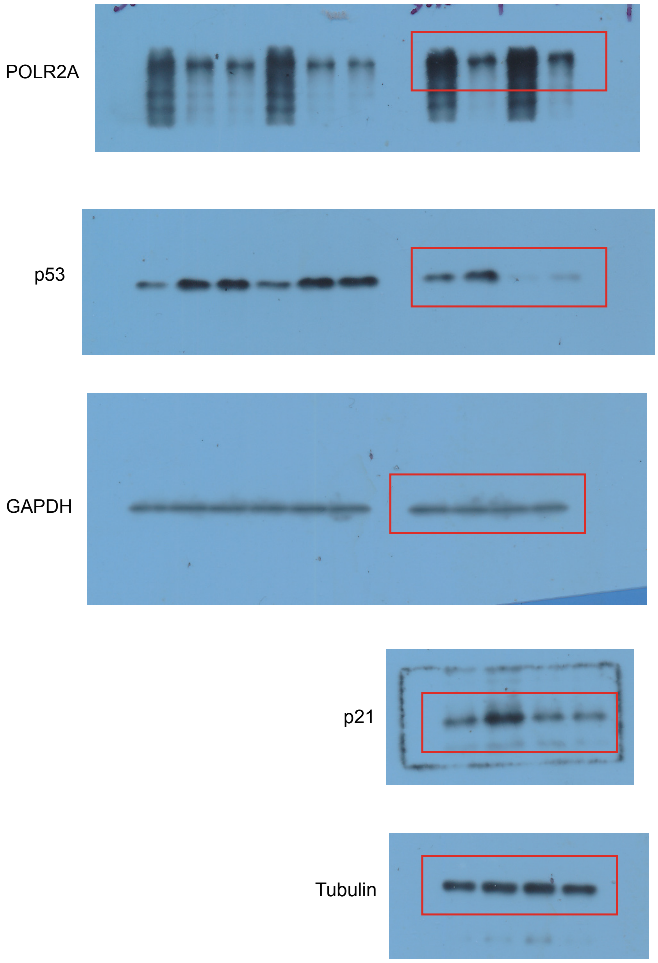
POLR2A
p53
GAPDH
p21
Tubulin

Figure 3

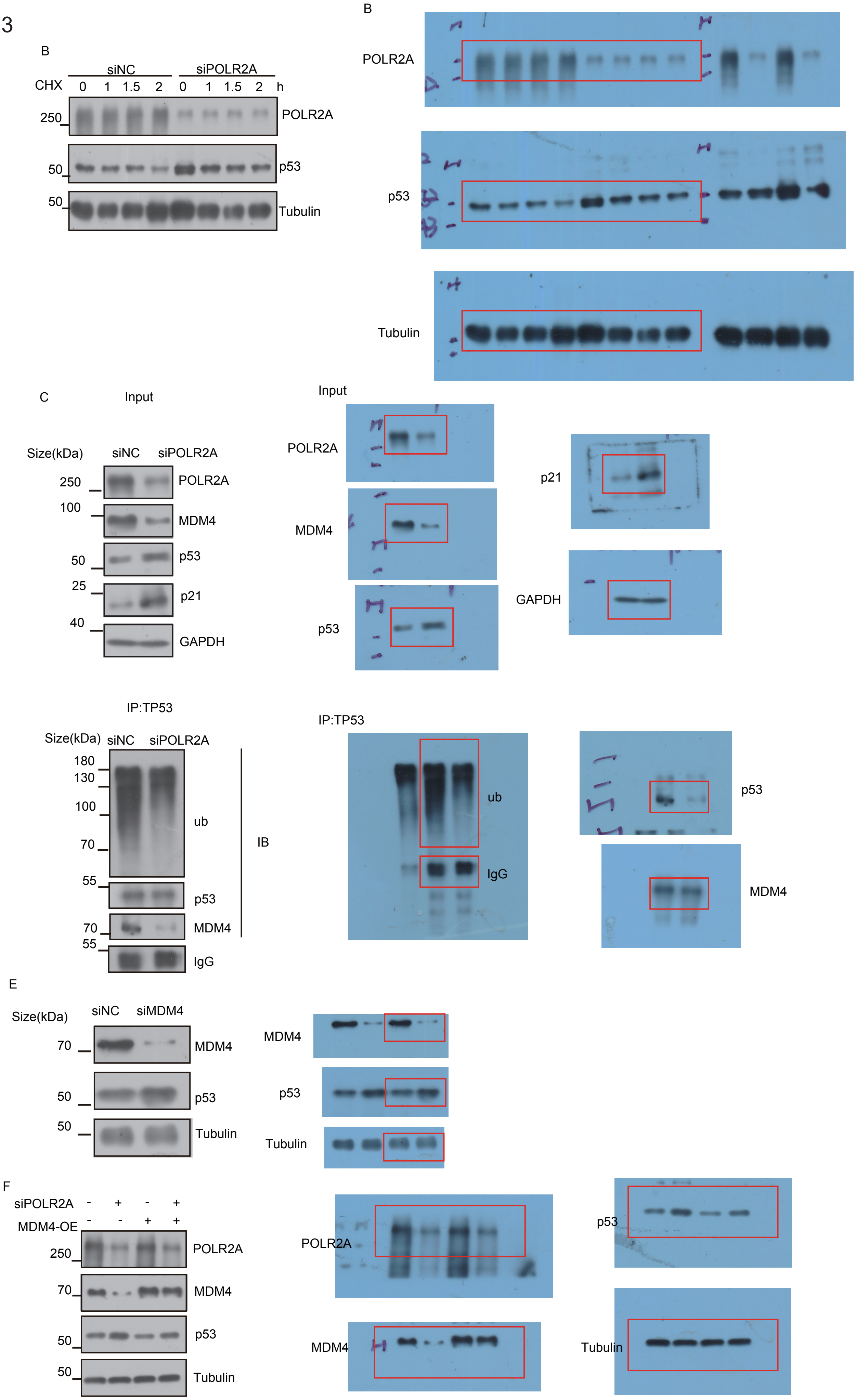
B
siNC
siPOLR2A
CHX 0 1 1.5 2 0 1 1.5 2 h
250
POLR2A
50
p53
50
Tubulin
B
POLR2A
p53
Tubulin
C
Input
Size(kDa) siNC siPOLR2A
250
POLR2A
100
MDM4
50
p53
25
p21
40
GAPDH
Input
POLR2A
MDM4
p53
p21
GAPDH
IP:TP53
Size(kDa) siNC siPOLR2A
180
130
100
ub
70
55
p53
70
MDM4
55
IgG
IB
IP:TP53
ub
IgG
p53
MDM4
E
Size(kDa) siNC siMDM4
70
MDM4
50
p53
50
Tubulin
MDM4
p53
Tubulin
F
siPOLR2A - + - +
MDM4-OE - - + +
250
POLR2A
70
MDM4
50
p53
50
Tubulin
POLR2A
MDM4
p53
Tubulin

Figure 4

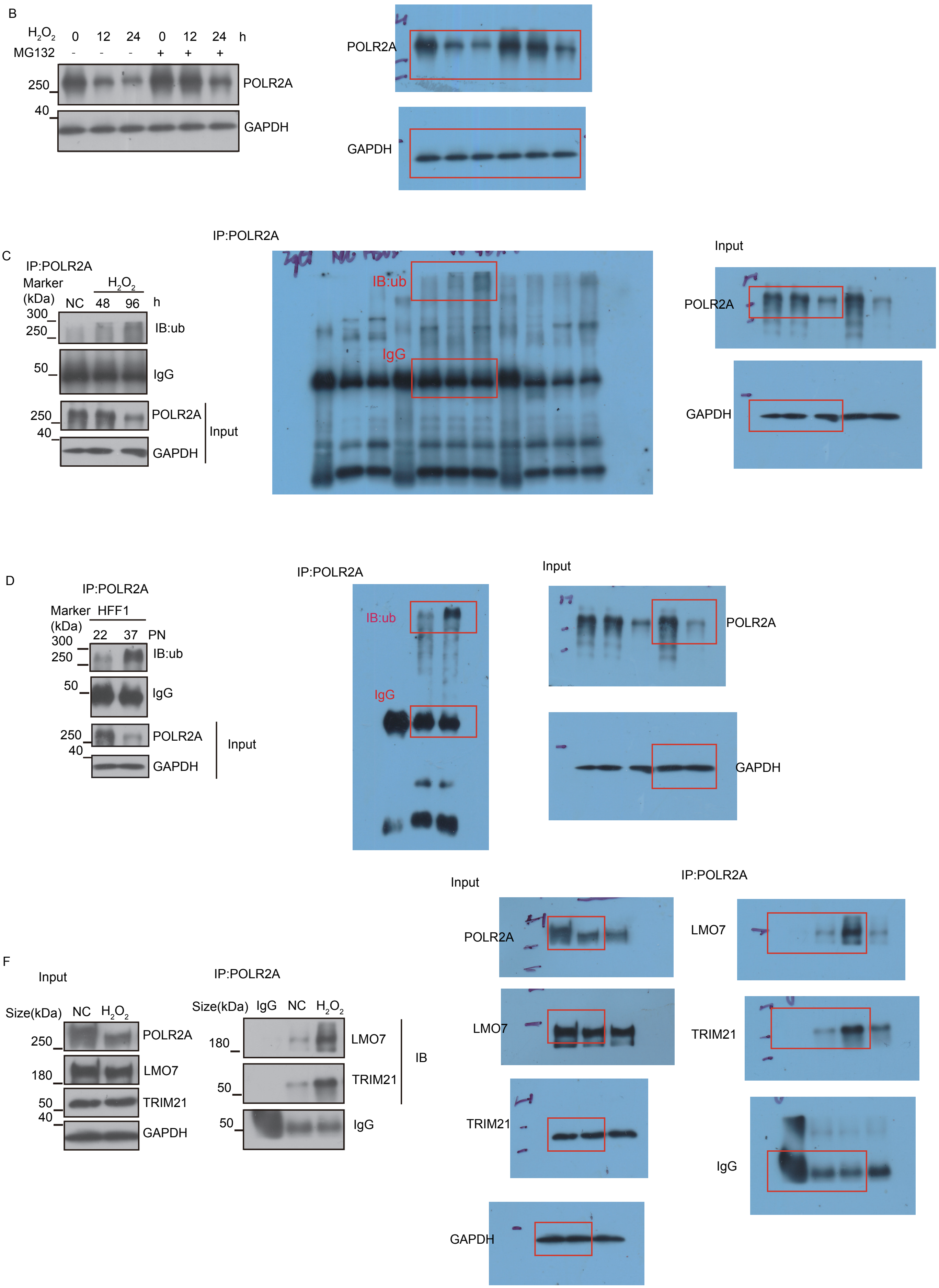

B
$H_2O_2$ 0 12 24 0 12 24 h
MG132 - - - + + +
250
40
POLR2A
GAPDH
POLR2A
GAPDH
C
IP:POLR2A
Marker (kDa)
NC 48 96 h
$H_2O_2$
300
250
IB:ub
50
IgG
250
40
POLR2A
GAPDH
Input
IP:POLR2A
IB:ub
IgG
Input
POLR2A
GAPDH
D
IP:POLR2A
Marker (kDa)
HFF1
22 37 PN
300
250
IB:ub
50
IgG
250
40
POLR2A
GAPDH
Input
IP:POLR2A
IB:ub
IgG
Input
POLR2A
GAPDH
F
Input
Size(kDa) NC $H_2O_2$
250
180
50
40
POLR2A
LMO7
TRIM21
GAPDH
IP:POLR2A
Size(kDa) IgG NC $H_2O_2$
180
50
50
LMO7
TRIM21
IgG
IB
Input
POLR2A
LMO7
TRIM21
GAPDH
IP:POLR2A
LMO7
TRIM21
IgG

Figure 4

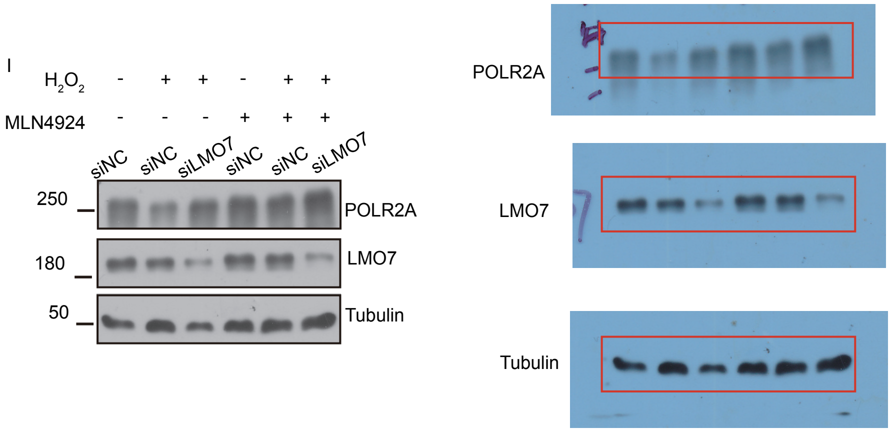
I
$H_2O_2$ - + + - + +
MLN4924 - - - + + +
siNC siNC siLMO7 siNC siNC siLMO7
250
POLR2A
180
LMO7
50
Tubulin
POLR2A
LMO7
Tubulin

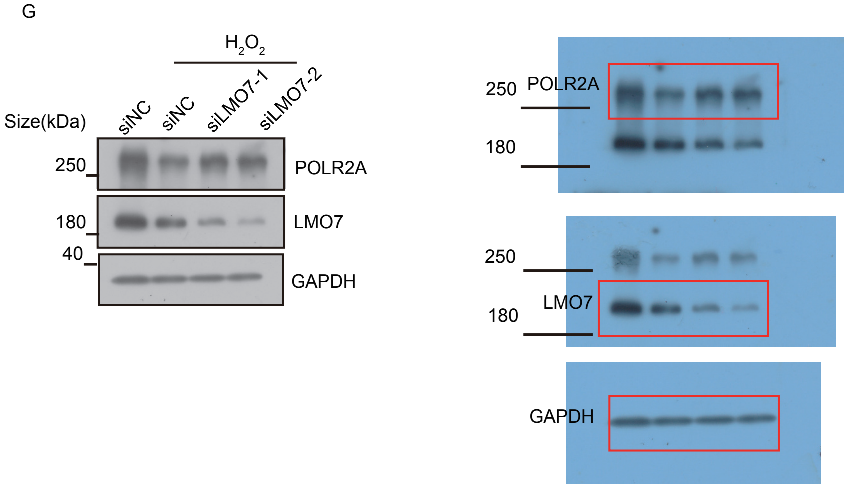
G
$H_2O_2$
Size(kDa)
siNC siNC siLMO7-1 siLMO7-2
250
POLR2A
180
LMO7
40
GAPDH
250
POLR2A
180
250
LMO7
180
GAPDH

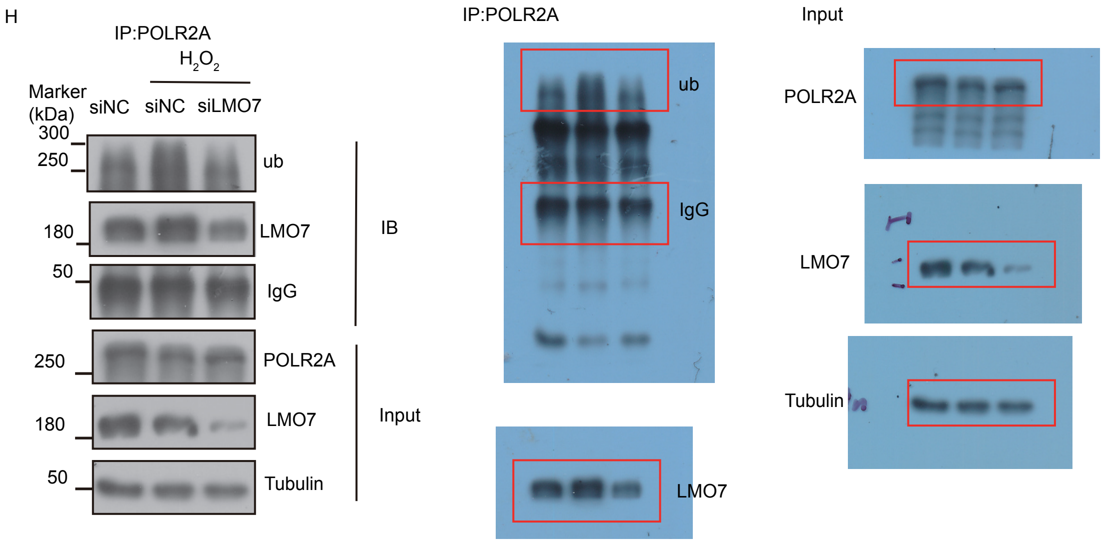
H
IP:POLR2A
$H_2O_2$
Marker (kDa)
siNC siNC siLMO7
300
250
ub
180
LMO7
50
IgG
IB
250
POLR2A
180
LMO7
50
Tubulin
Input
IP:POLR2A
ub
IgG
LMO7
Input
POLR2A
LMO7
Tubulin

# FIGURE5

A

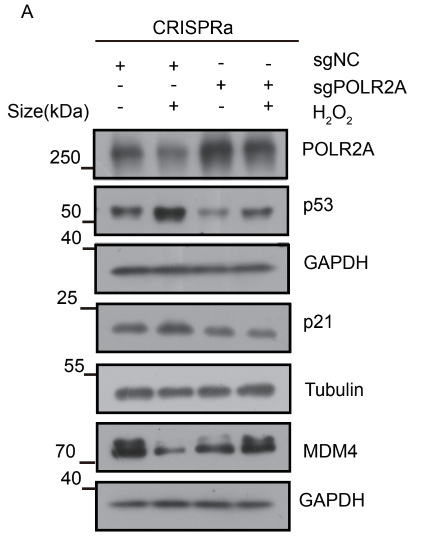

CRISPRa
sgNC
sgPOLR2A
H2O2
Size(kDa)
250
50
40
25
55
70
40
POLR2A
p53
GAPDH
p21
Tubulin
MDM4
GAPDH


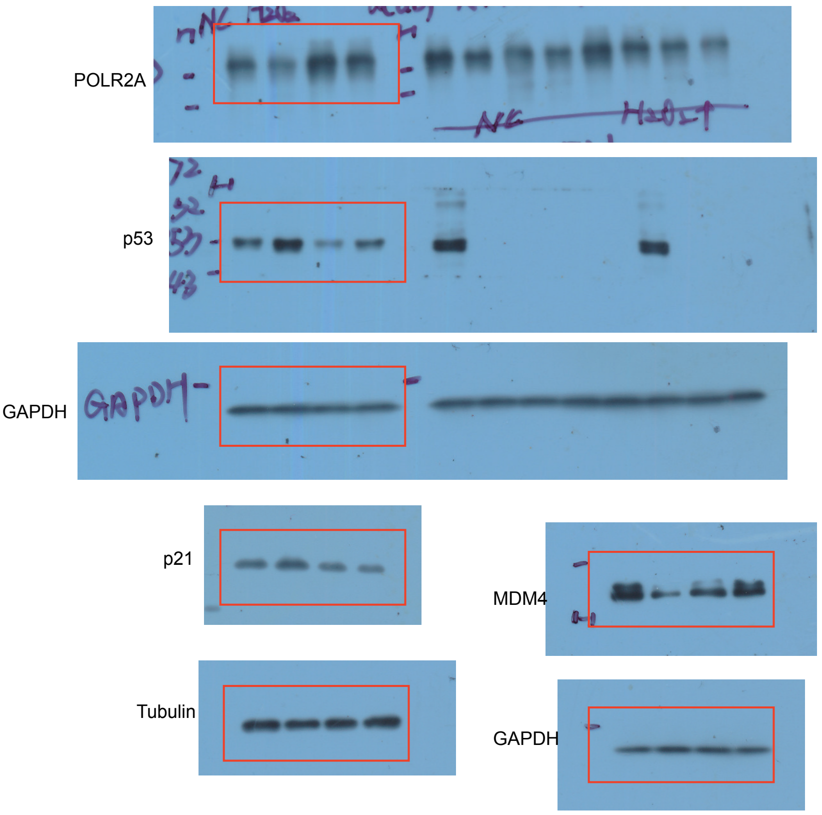

POLR2A
p53
GAPDH
p21
Tubulin
MDM4
GAPDH


D

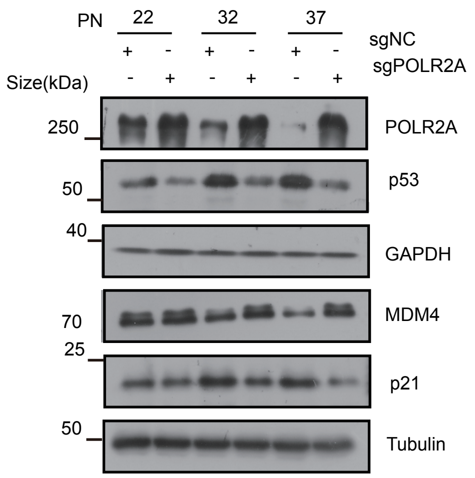

PN
22
32
37
sgNC
sgPOLR2A
Size(kDa)
250
50
40
70
25
50
POLR2A
p53
GAPDH
MDM4
p21
Tubulin


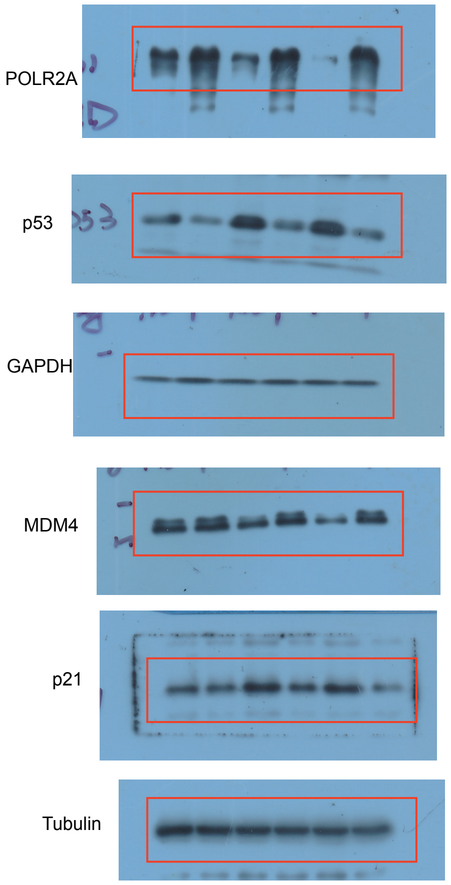

POLR2A
p53
GAPDH
MDM4
p21
Tubulin

Supplementary Fig. 1

B

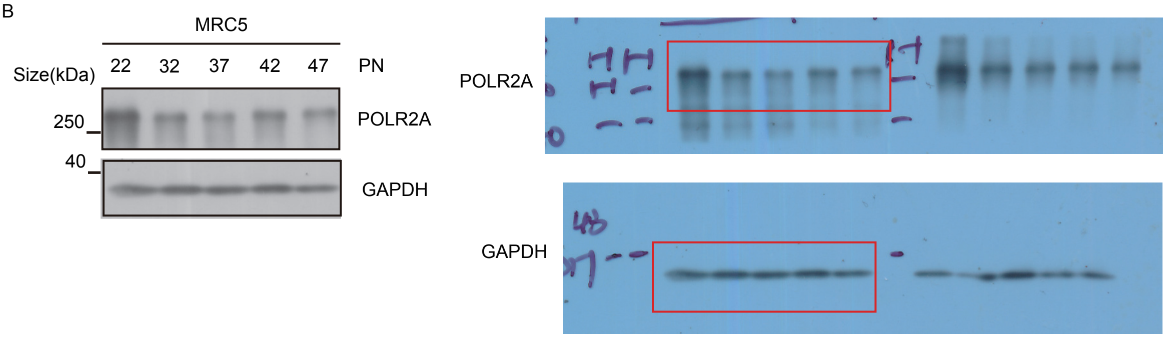


D

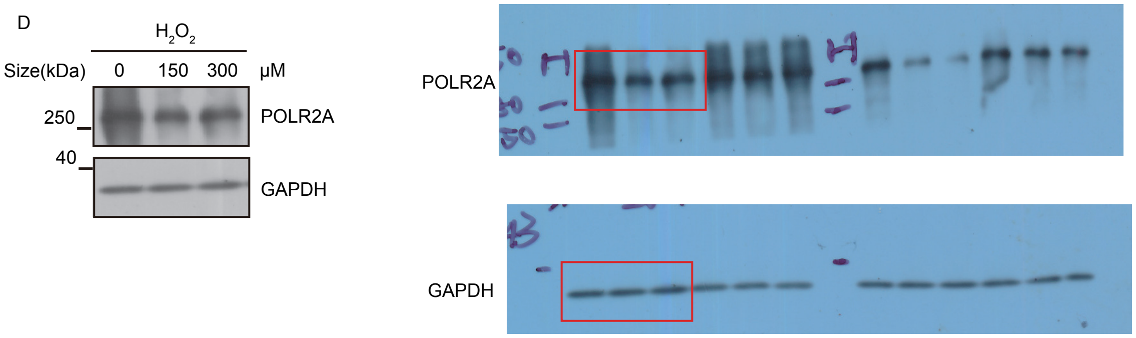

Supplementary Fig. 3

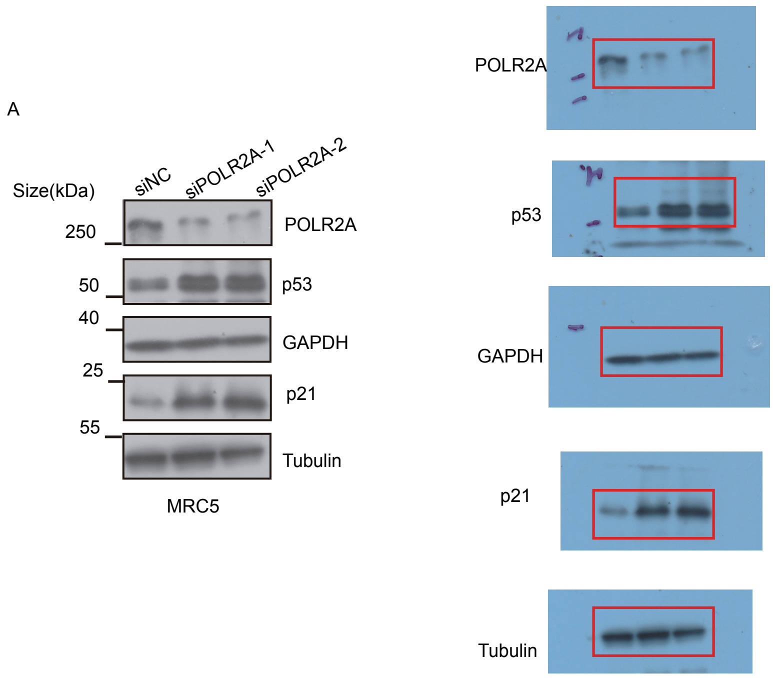

Supplementary Fig. 4

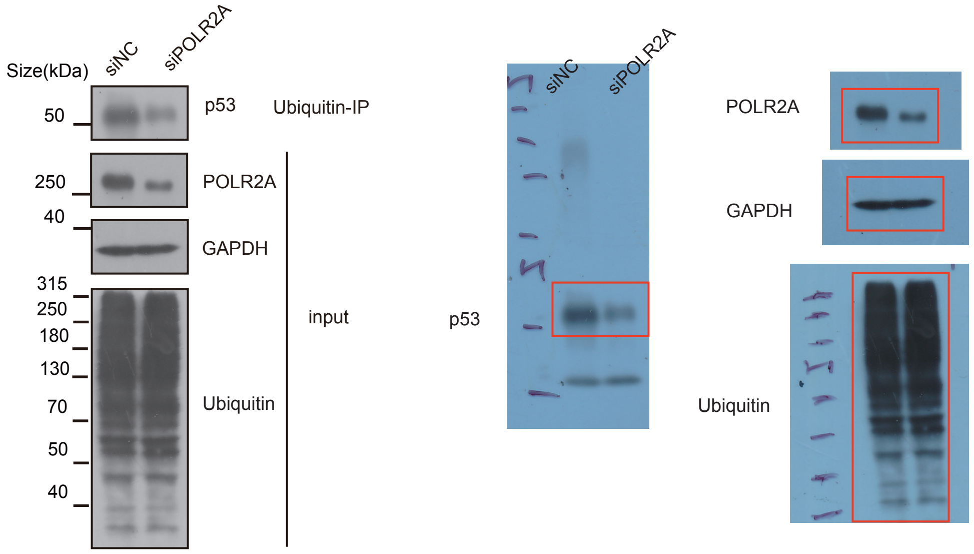

Supplementary Fig. 5

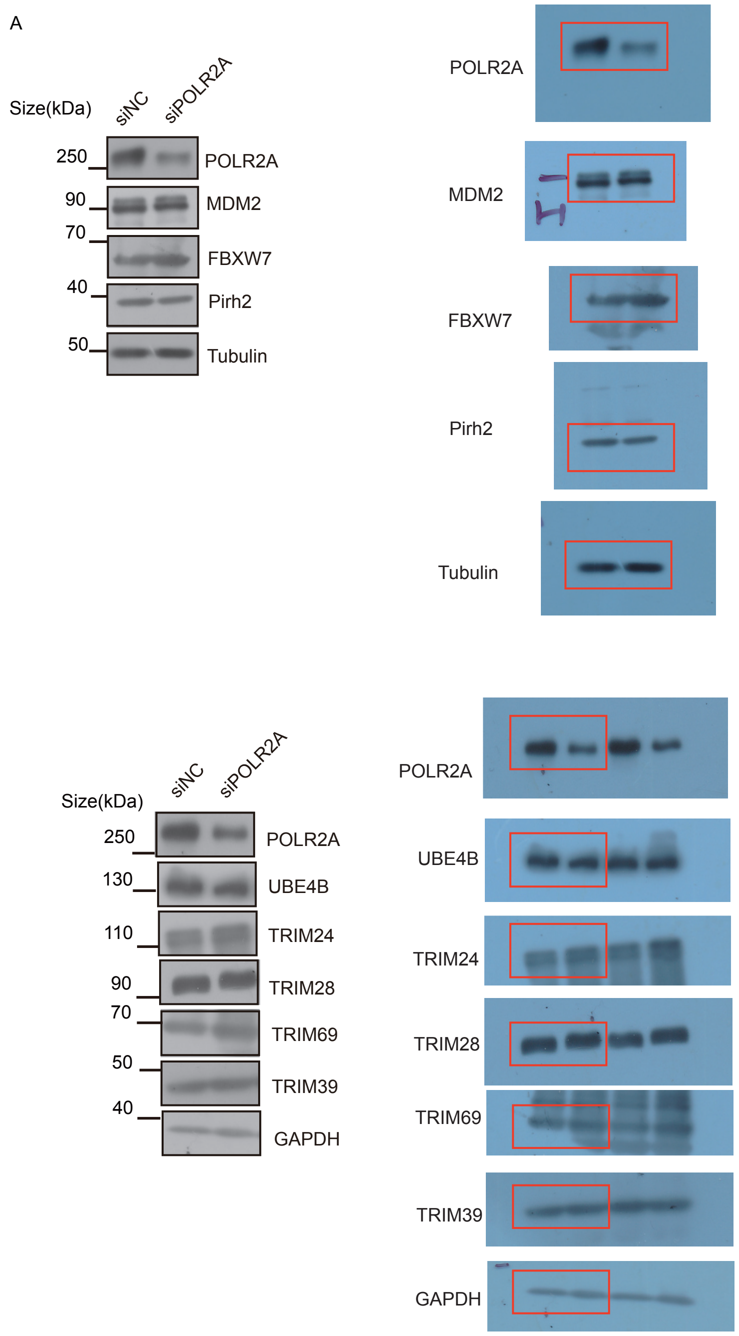

Supplementary Fig. 5

B

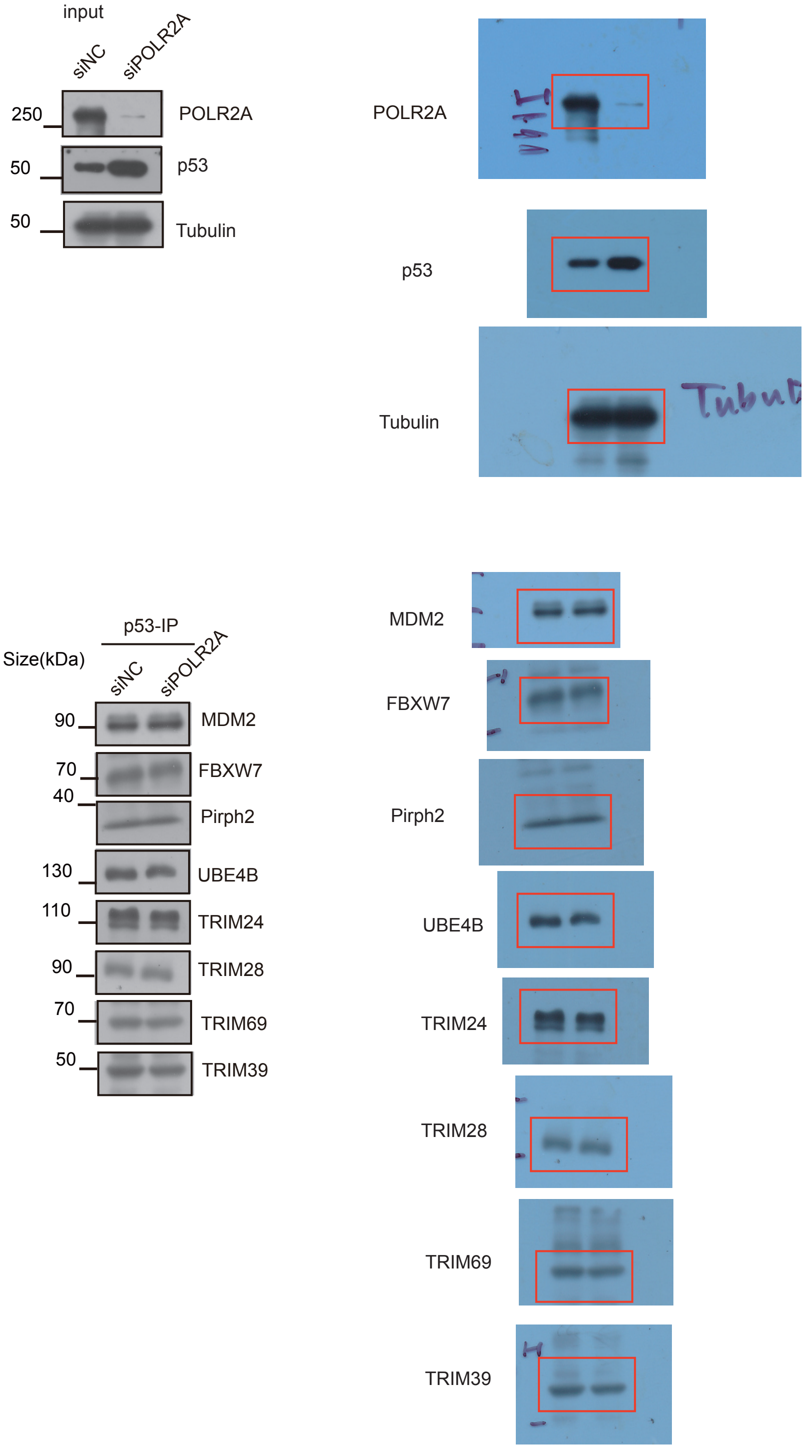

Supplementary Fig. 6

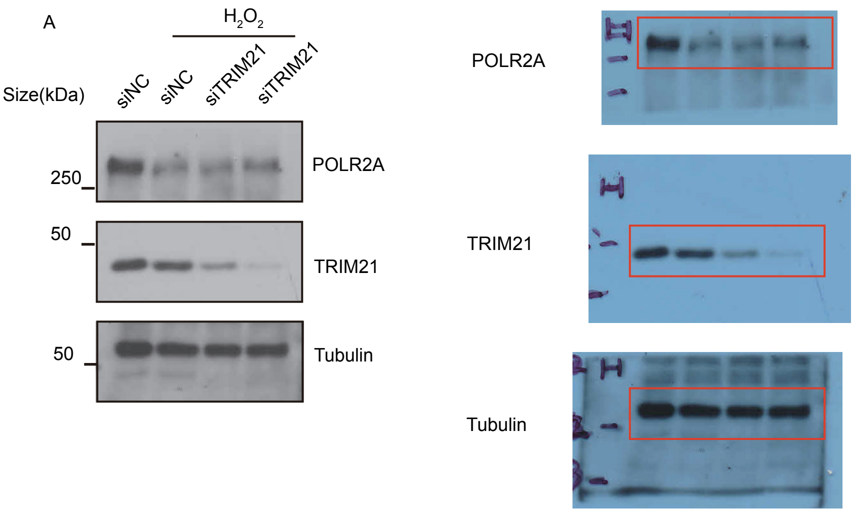

Supplement: Supplementary file 15 — Original Data [file 41419_2026_8679_MOESM15_ESM.pdf]
